# Supplementary material for: Population reconstructions for humans and megafauna suggest mixed causes for North American Pleistocene extinctions
Source: Nat Commun. 2018 Dec 21;9:5441. doi: 10.1038/s41467-018-07897-1 (PMC6303330; doi:10.1038/s41467-018-07897-1)
Supplement: Supplementary file 1 — Supplementary Information [file 41467_2018_7897_MOESM1_ESM.pdf]

**Supplementary Information**

# Population reconstructions for humans and megafauna suggest mixed causes for North American Pleistocene Extinctions

Broughton and Weitzel

**Supplementary Note 1: Analysis of Vetted Pre-Clovis Dataset**

We present here analyses of the radiocarbon records from the contiguous US as in the main text, but use for the pre-Clovis period (> 13.2 ka) only dates from those sites that are widely accepted as providing the most secure evidence of human occupation: Paisley Caves, Meadowcroft Rockshelter, and Page-Ladson[1-4]. As a large number of radiocarbon assays have been generated from non-cultural materials from these sites, we used only dates that were derived from artifacts, features, or human remains, or from materials determined by the investigators to be in close association with the latter (Supplementary Table 1). Using this vetted pre-Clovis dataset, Supplementary Figure 1 shows the SPDs for the human and various megafaunal taxa for the contiguous US. The overall trends, periods of significant decline, and general conclusions closely match those obtained using the larger CARD data set (Supplementary Table 2).

**Supplementary Note 2: Analysis of Taphonomically Corrected Data**

The following analysis is based on the human and megafaunal SPDs after applying the taphonomic correction equation described in Surovell *et al.*[5]. This equation is based on comparisons between the known global history of volcanic activity as recorded from ice core records (GISP2) and the radiocarbon record of terrestrial volcanic deposits. Periods with high known volcanic activity, for example, that lack corresponding terrestrial volcanic records reflect periods where geologically-based taphonomic loss or bias has occurred. The method assumes such losses would also apply to archaeological or paleontological materials for those same times and adjusts date frequencies accordingly. Supplementary Figures 2-4 show the SPDs for the human and various megafaunal taxa. The overall trends, periods of significant decline, and general conclusions are nearly identical to those derived from the analyses of the observed data (Supplementary Figure 5; Supplementary Table 3).

**Supplementary Note 3: Analysis Using Logistic Null Models**

Supplementary Figures 6-8 show the SPDs for the human and various megafaunal taxa and statistically significant deviations using logistic, rather than exponential, null models. The results shown here for all regions and taxa are in almost all cases very similar to the analyses conducted with exponential null models with notable differences occurring only with US mammoth, US mastodon, and US *Equus*. In the case of US mammoth, a brief return to trend occurs during the early YD after a brief bust toward the end of the Clovis period. The terminal decline for mammoth thus occurs during the YD rather than in Clovis times as indicated in the analysis based on an exponential model. The decline during the Clovis period is still evident, however, and given the significant correlation with the human SPD is in any case consistent with a human role in mammoth extinction. For US mastodon, the terminal decline date shifts into the Clovis period from its place in the YD with the exponential null model. However, since the mastodon and human SPDs do not exhibit a meaningful correlation, the difference in this case pertains only to the timing of the apparent climatic influences on this taxon—effectively pushing the terminal decline back ~400 years. Finally, for US *Equus*, there are several pre-Clovis “busts” while the overall trend of the SPD is still increasing. This is due to a poorly-fit logistic null model with a top asymptote far above the highest SPD value. When simulating the logistic null model confidence interval, this has the effect of dragging the interval upwards for this portion of the curve, resulting in the discrepancy observed here. However, this result is due to a poorly fit model and should be treated with skepticism: it is quite clear from visual inspection of this SPD that such “busts” occur during a period of general increase and are therefore not related to declines towards extinction. In sum, the overall trends and general conclusions regarding the causative factors relating to megafaunal extinctions are robust to the use of either exponential or logistic null models.

Supplementary Table 1. Radiocarbon determinations from Page- Ladson, Meadowcroft Rockshelter, and Paisley Caves.

| Site | Lab No | Uncorrected Date | One sigma | OxCal Old | OxCal Young | OxCal Median | Material | Reference |
|---|---|---|---|---|---|---|---|---|
| Page-Ladson | UCIAMS-141949 | 12,385 | 35 | 14725 | 14164 | 14421 | *Quercus* sp. twig | 1 |
| Page-Ladson | UCIAMS-127302 | 12,425 | 30 | 14818 | 14220 | 14520 | *Acer* (wood) | 1 |
| Page-Ladson | UCIAMS-127304 | 12,420 | 30 | 14801 | 14212 | 14508 | *Abies* (wood) | 1 |
| Page-Ladson | UCIAMS-127305 | 13,015 | 30 | 15762 | 15357 | 15587 | unknown periderm (bark) | 1 |
| Page-Ladson | UCIAMS-127303 | 12,495 | 30 | 15042 | 14381 | 14737 | *Fraxinus* sp. (wood) | 1 |
| Page-Ladson | UCIAMS-141950 | 13,830 | 35 | 16959 | 16516 | 16738 | *Taxodium* (wood) | 1 |
| Page-Ladson | UCIAMS-141951 | 12,415 | 30 | 14786 | 14206 | 14495 | *Leucobalanus* (wood) | 1 |
| Page-Ladson | UCIAMS-143535 | 12,420 | 30 | 14801 | 14212 | 14508 | unidentified hardwood (wood) | 1 |
| Page-Ladson | UCIAMS-143536 | 12,360 | 35 | 14676 | 14136 | 14360 | unidentified hardwood (wood) | 1 |
| Page-Ladson | UCIAMS-143537 | 12,560 | 35 | 15125 | 14664 | 14902 | unidentified hardwood (wood) | 1 |
| Page-Ladson | UCIAMS-143538 | 12,430 | 35 | 14852 | 14220 | 14533 | unidentified hardwood (wood) | 1 |
| Meadowcroft | SI-2491 | 11,300 | 700 | 15372 | 11328 | 13292 | charcoal | 2 |
| Meadowcroft | SI-2489 | 12,800 | 870 | 17796 | 13165 | 15318 | charcoal | 2 |
| Meadowcroft | SI-2065 | 13,240 | 1010 | 18700 | 13377 | 15948 | charcoal | 2 |
| Meadowcroft | SI-2488 | 13,270 | 340 | 17064 | 14948 | 15948 | charcoal | 2 |
| Meadowcroft | SI-1872 | 14,975 | 620 | 19750 | 16640 | 18208 | charcoal | 2 |
| Meadowcroft | SI-1686 | 15,120 | 165 | 18723 | 17978 | 18367 | charcoal | 2 |
| Meadowcroft | SI-2354 | 16,175 | 975 | 22339 | 17576 | 19726 | charcoal | 2 |
| Paisley Caves | UCIAMS-77104 | 11,625 | 35 | 13565 | 13383 | 13457 | human coprolite | 3-4 |
| Paisley Caves | UCIAMS-90593 | 11,930 | 25 | 13954 | 13570 | 13756 | butcher-cut bone | 3-4 |
| Paisley Caves | UCIAMS-90581 | 11,340 | 30 | 13273 | 13100 | 13183 | human coprolite | 3-4 |
| Paisley Caves | QxA-16495 | 12,140 | 70 | 14186 | 13779 | 14009 | human coprolite | 3-4 |
| Paisley Caves | UCIAMS-79706 | 12,165 | 25 | 14160 | 13954 | 14057 | human coprolite | 3-4 |
| Paisley Caves | UCIAMS-68017 | 12,195 | 30 | 14199 | 13977 | 14086 | modified sawtooth bear bone | 3-4 |
| Paisley Caves | UCIAMS-76190 | 12,265 | 25 | 14305 | 14048 | 14163 | human coprolite | 3-4 |
| Paisley Caves | OxA-16498 | 12,275 | 55 | 14546 | 14008 | 14199 | human coprolite | 3-4 |
| Paisley Caves | OxA-16497 | 12,345 | 55 | 14711 | 14101 | 14352 | human coprolite | 3-4 |

Supplementary Table 2. Rank order correlations between human and megafauna SPDs for the vetted pre-Clovis dataset.

| Comparison | Rho | Date (cal BP) of terminal bust |
|---|---|---|
| Mammoth-human | -0.67* | 13,006 |
| Mastodon-human | -0.15* | 12,638 |
| S. ground sloth-human | 0.21* | 12,654 |
| Horse-human | -0.48* | 12,947 |
| Saber-toothed cat-human | -0.80* | 13,032 |

* P-values < 0.0001

Supplementary Table 3. Rank order correlations between humans and megafauna SPDs for the taphonomically corrected datasets.

| Comparison | Region | Rho | Date (cal BP) of terminal bust for megafauna |
|---|---|---|---|
| Mammoth-human | Cont. US | -0.67* | 13,020 |
| Mastodon-human | Cont. US | -0.04** | 12,679 |
| S. ground sloth-human | Cont. US | 0.29* | 12,660 |
| Horse-human | Cont. US | -0.43* | 12,958 |
| Saber-toothed cat-human | Cont. US | -0.68* | 13,048 |
| | | | |
| S. ground sloth-human | Southwest | 0.58* | 12,687 |
| Mammoth-human | Southwest | -0.78* | 12,553 |
| | | | |
| Mastodon-human | Great Lakes | 0.58* | 12,588 |
| Mammoth-human | Great Lakes | 0.25* | 12,012 |

* P-values < 0.0001

** P-value = 0.03

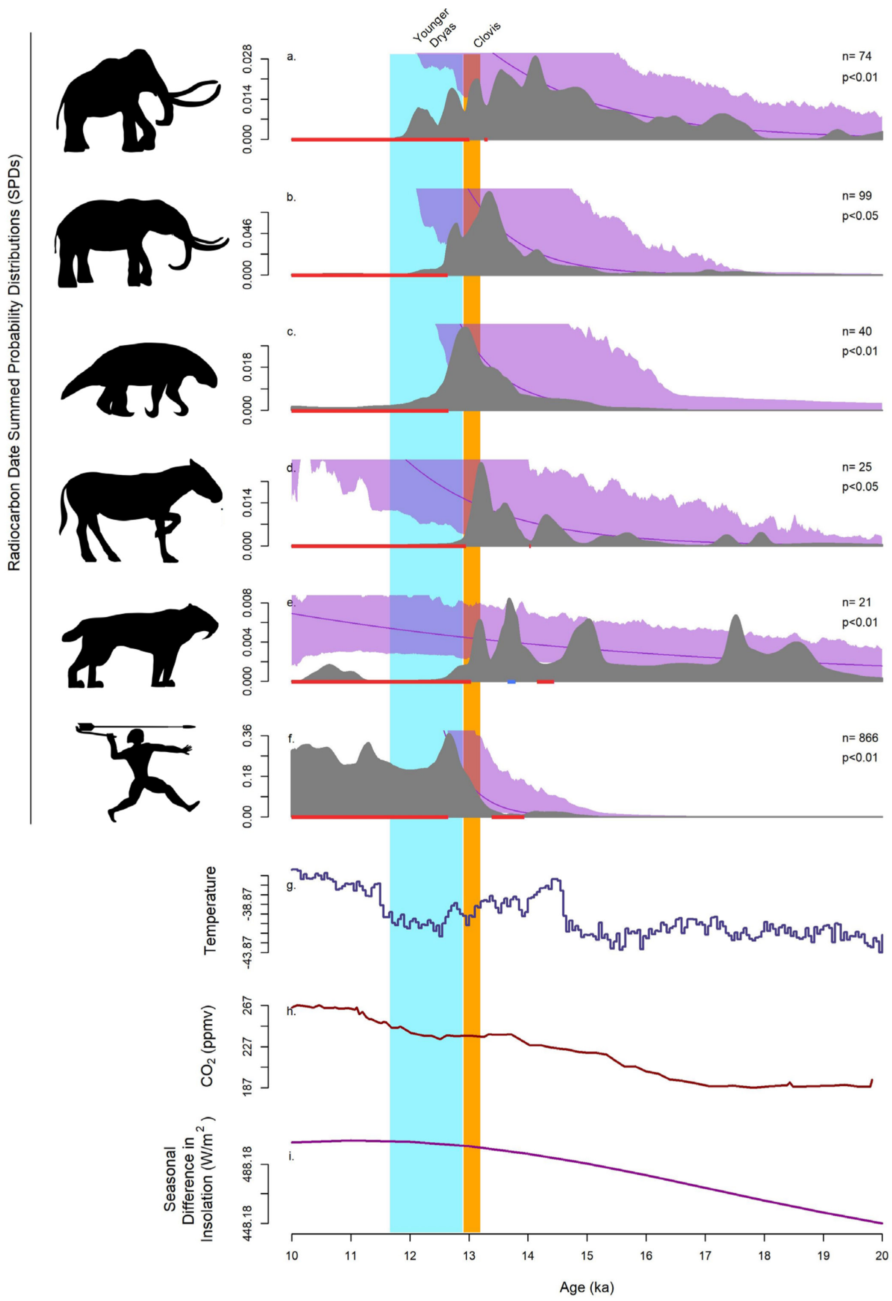


Supplementary Figure 1. SPDs for mammoth (a), mastodon (b), Shasta ground sloth (c), horse (d), saber-toothed cat (e), and human populations (f) through time for the contiguous United States are indicated in gray based on the vetted pre-Clovis dataset. The null model of exponential growth is indicated by the purple line; the purple shading denotes the 95% confidence interval around the null model (see Methods). Statistically significant deviations from the null model are indicated by the red (busts) and blue (booms) rugs at the base of each panel. Time series of $\delta^{18}O$ values from the NGRIP ice core (g), $CO_2$ from the Dome C, Antarctica, ice core (h), and insolation seasonality (i) are provided for comparison. Data sources are in Methods.

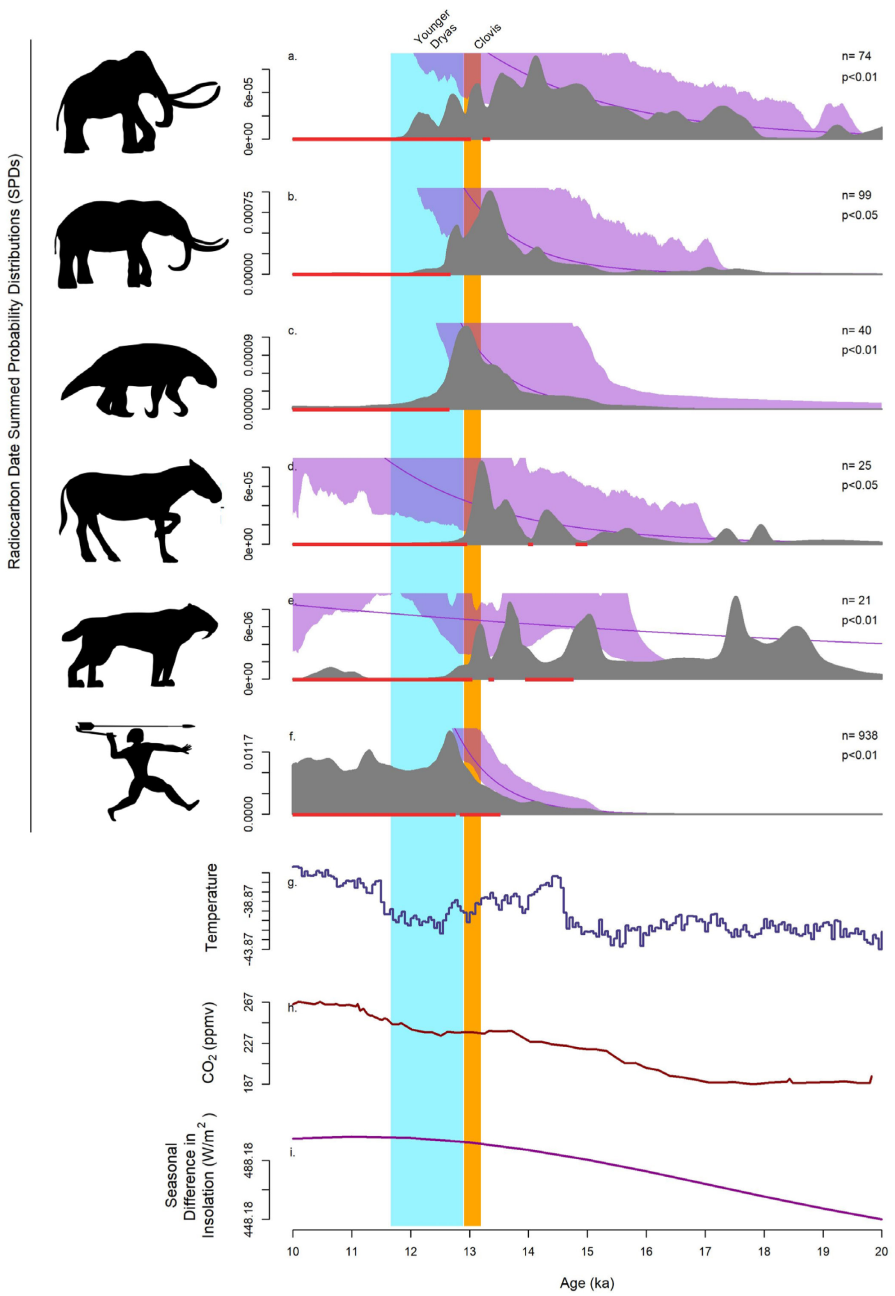


Supplementary Figure 2. SPDs based on the taphonomically corrected data for mammoth (a), mastodon (b), Shasta ground sloth (c), horse (d), saber-toothed cat (e), and human populations (f) through time for the contiguous United States are indicated in gray. The null model of exponential growth is indicated by the purple line; the purple shading denotes the 95% confidence interval around the null model (see Methods). Statistically significant deviations from the null model are indicated by the red (busts) and blue (booms) rugs at the base of each panel. Time series of $\delta^{18}O$ values from the NGRIP ice core (g), $CO_2$ from the Dome C, Antarctica, ice core (h), and insolation seasonality (i) are provided for comparison. Data sources are in Methods.

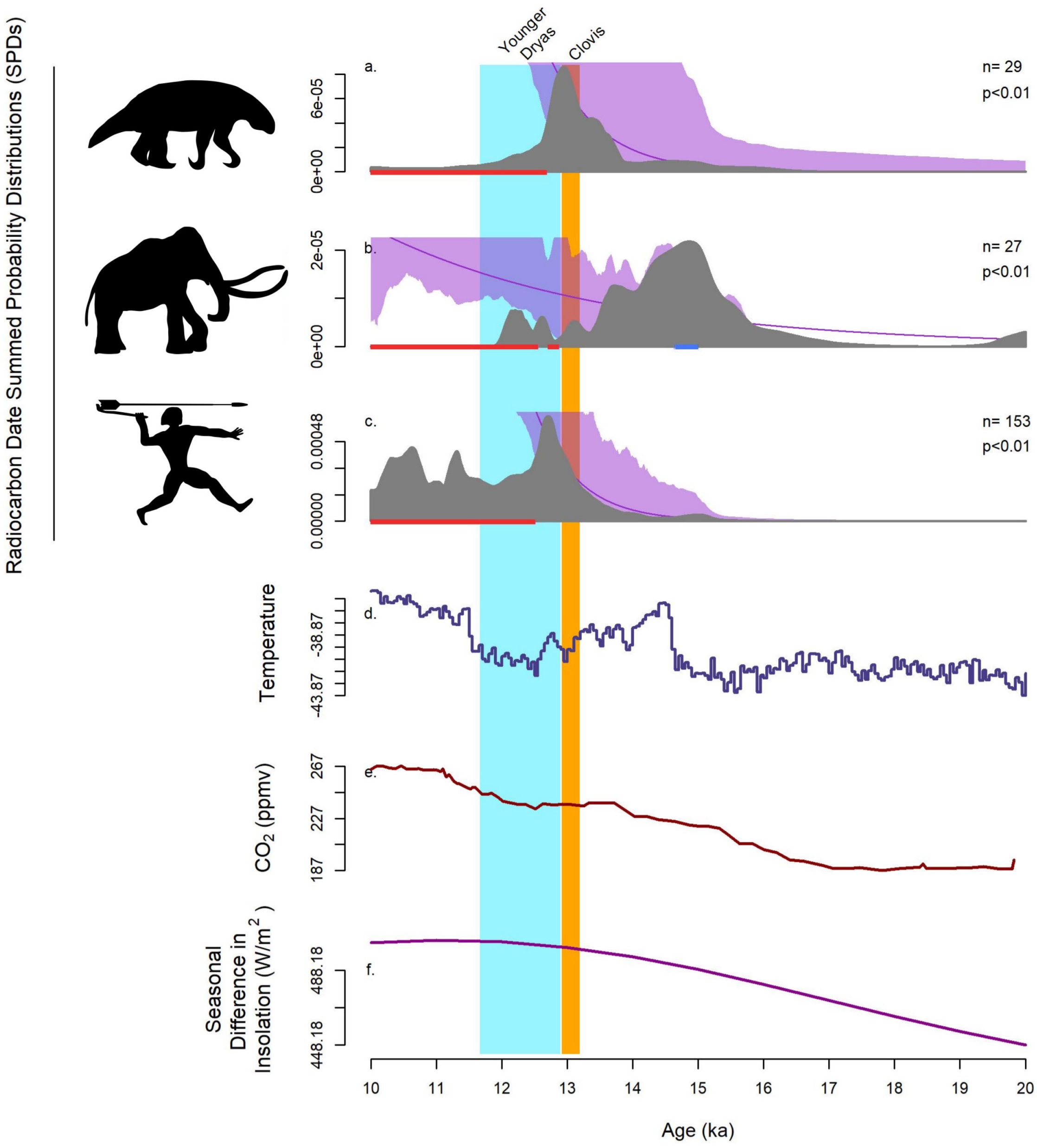


Supplementary Figure 3. SPDs based on the taphonomically corrected data for Shasta ground sloth (a), mammoth (b), and human populations (c) through time for the Southwest region are indicated in gray. The null model of exponential growth is indicated by the purple line; the purple shading denotes the 95% confidence interval around the null model (see Methods). Statistically significant deviations from the null model are indicated by the red (busts) and blue (booms) rugs at the base of each panel. Time series of $\delta^{18}O$ values from the NGRIP ice core (d), $CO_2$ from the Dome C, Antarctica, ice core (e), and insolation seasonality (f) are provided for comparison. Data sources are in Methods.

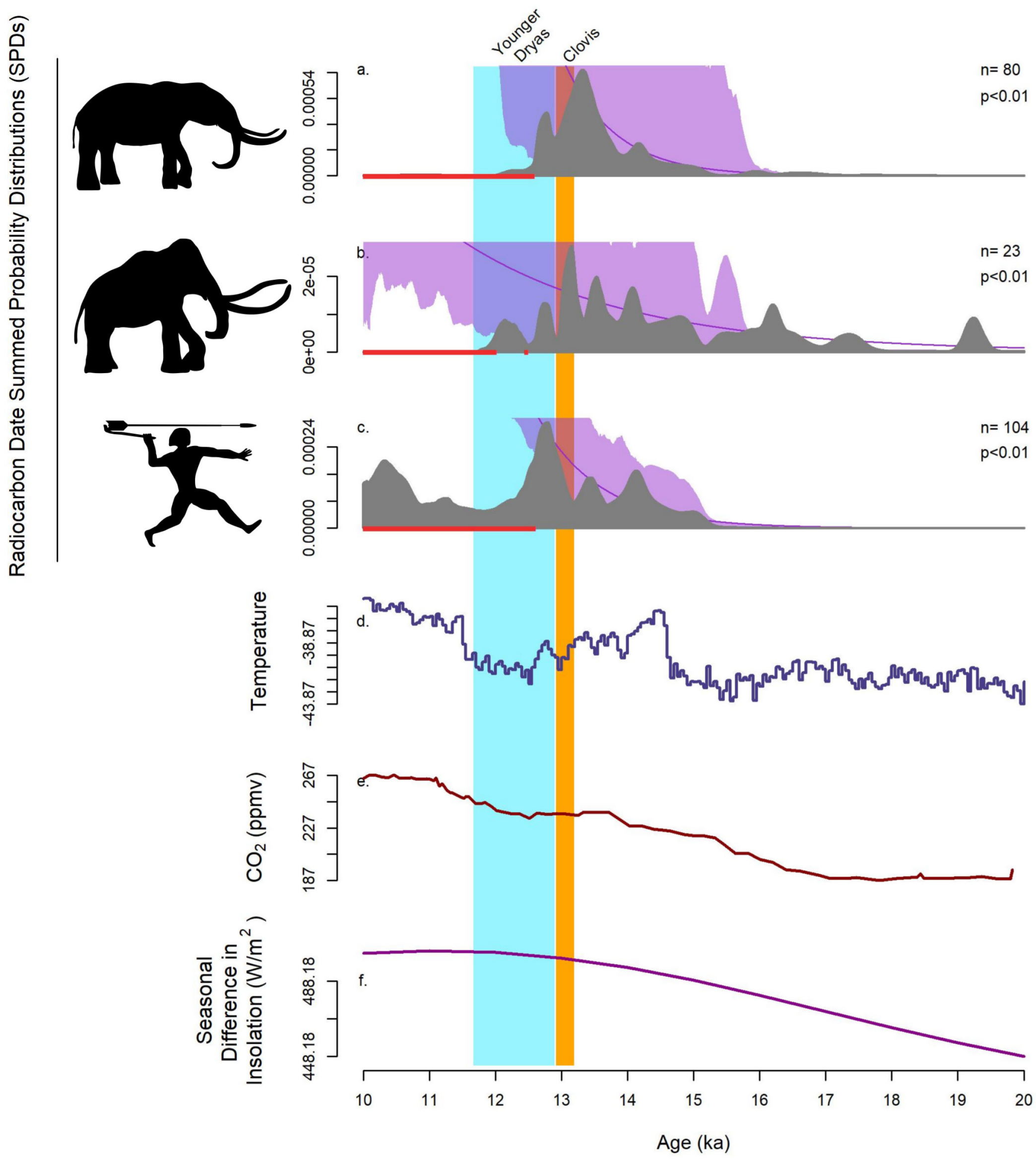


Supplementary Figure 4. SPDs based on the taphonomically corrected data for mastodon (a), mammoth (b), and human populations (c) through time for the Great Lakes region are indicated in gray. The null model of exponential growth is indicated by the purple line; the purple shading denotes the 95% confidence interval around the null model (see Methods). Statistically significant deviations from the null model are indicated by the red (busts) and blue (booms) rugs at the base of each panel. Time series of $\delta^{18}O$ values from the NGRIP ice core (d), $CO_2$ from the Dome C, Antarctica, ice core (e), and insolation seasonality (f) are provided for comparison. Data sources are in Methods.

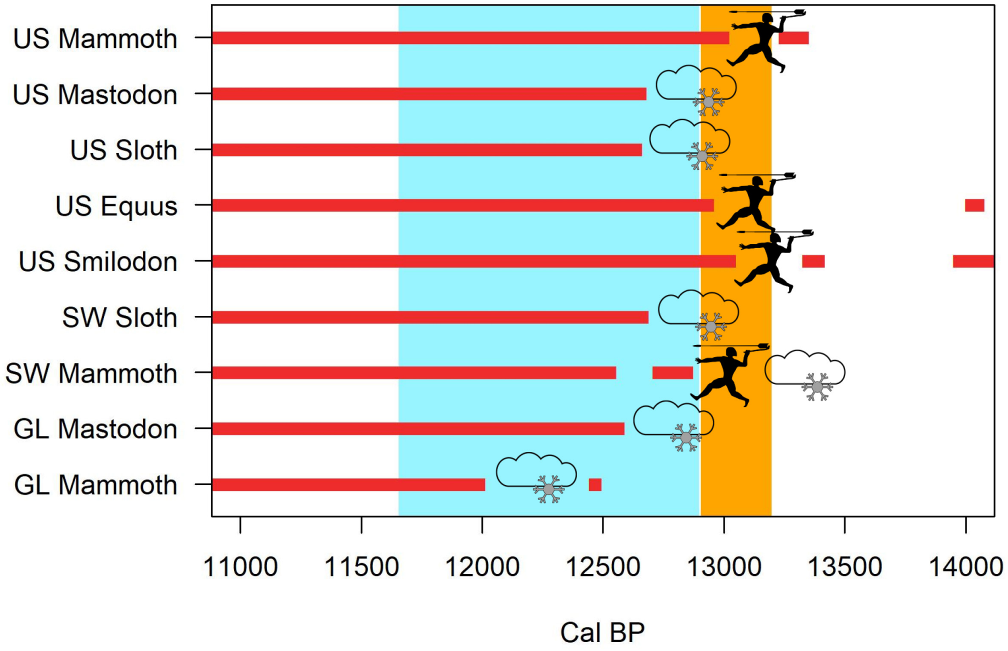


Supplementary Figure 5. Summary of the chronology for megafaunal population busts by region in relation to the Clovis period and the Younger Dryas based on the taphonomically corrected data. Symbols to the right indicate extinction cause(s) suggested by this analysis: clouds and snowflake = climate; human with spear = hunting.

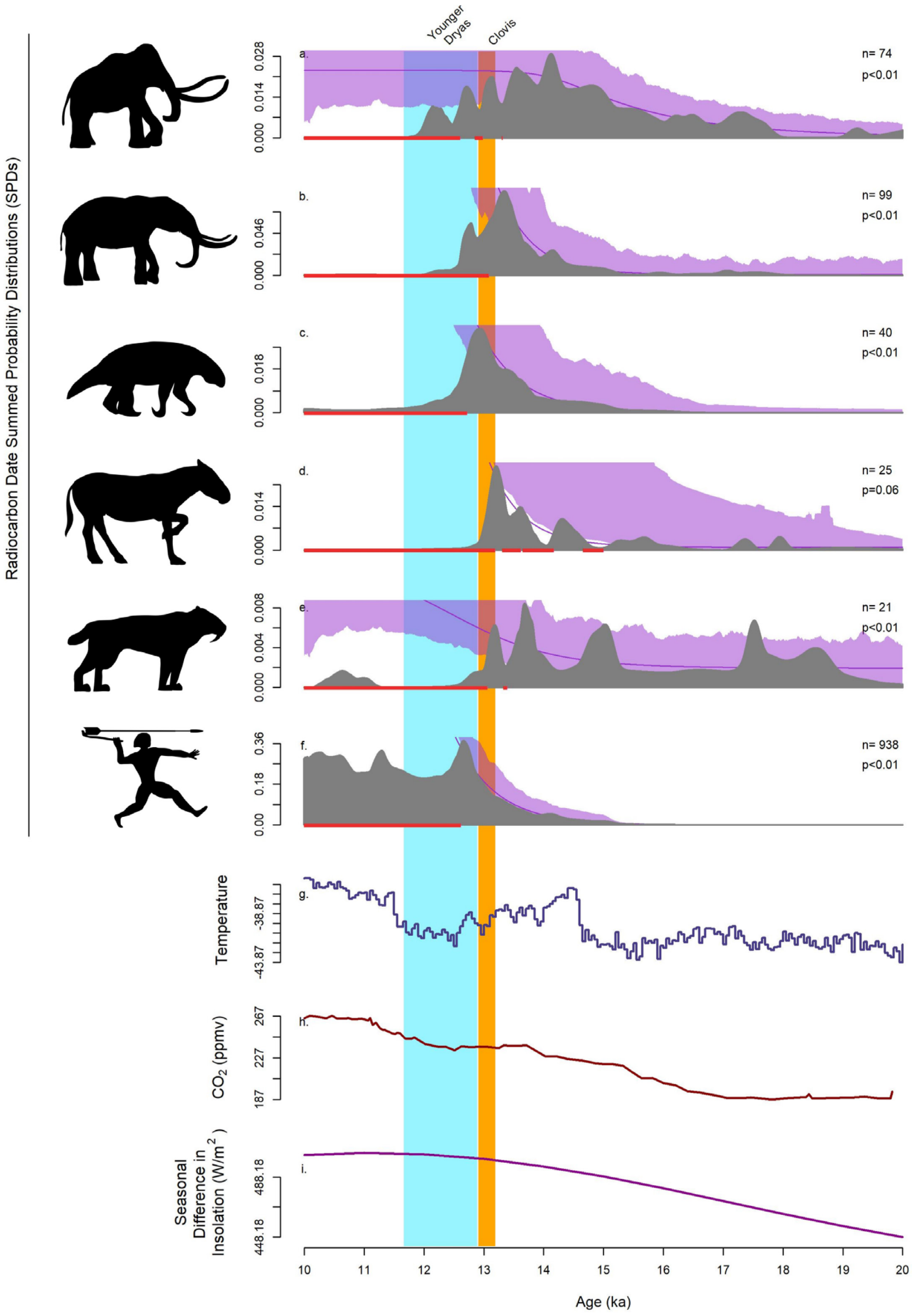


Supplementary Figure 6. SPDs for mammoth (a), mastodon (b), Shasta ground sloth (c), horse (d), saber-toothed cat (e), and human populations (f) through time for the contiguous United States are indicated in gray. The null model of logistic growth is indicated by the purple line; the purple shading denotes the 95% confidence interval around the null model (see Methods). Statistically significant deviations from the null model are indicated by the red (busts) and blue (booms) rugs at the base of each panel. Time series of $\delta^{18}O$ values from the NGRIP ice core (g), $CO_2$ from the Dome C, Antarctica, ice core (h), and insolation seasonality (i) are provided for comparison. Data sources are in Methods.

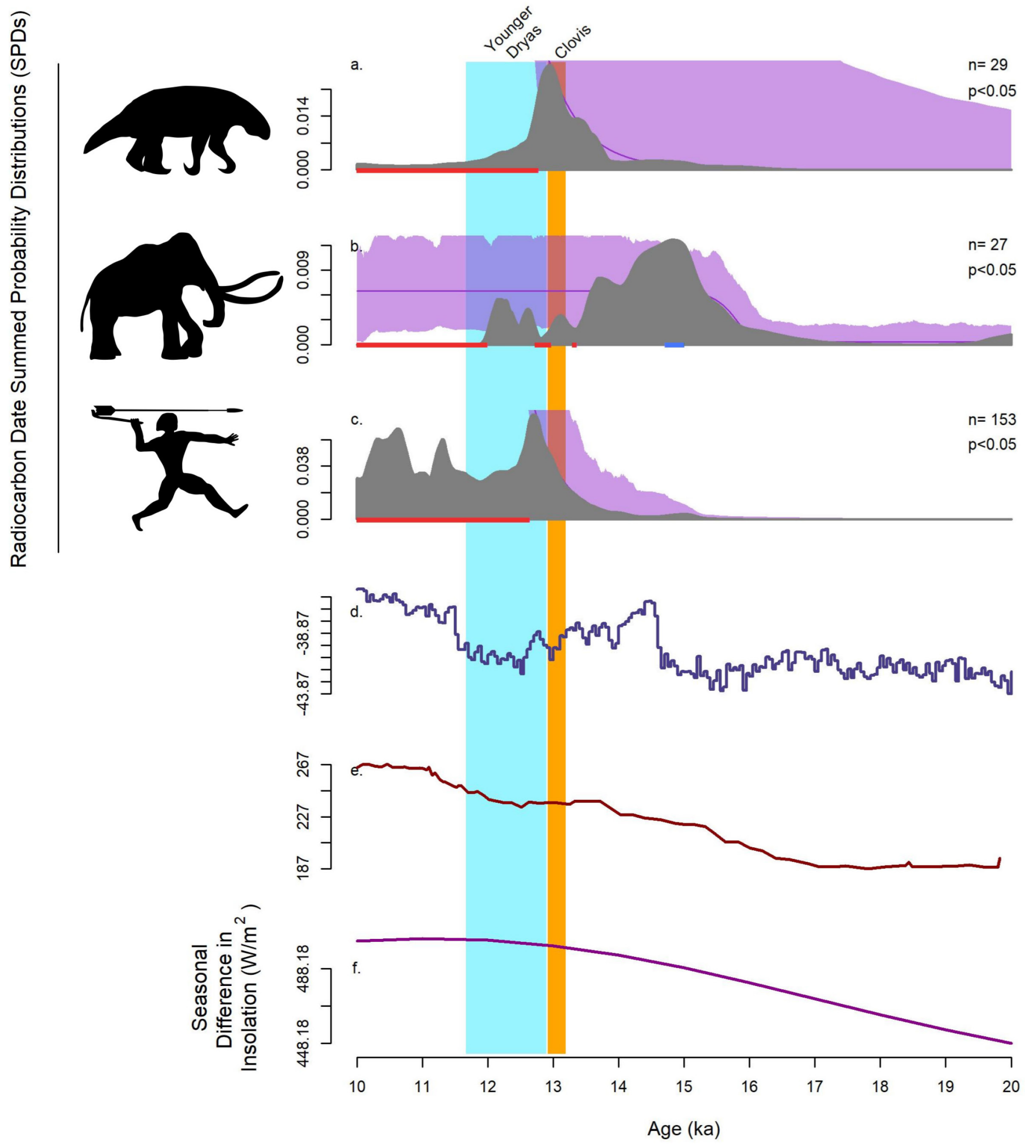


Supplementary Figure 7. SPDs for Shasta ground sloth (a), mammoth (b), and human populations (c) through time for the Southwest are indicated in gray. The null model of logistic growth is indicated by the purple line; the purple shading denotes the 95% confidence interval around the null model (see Methods). Statistically significant deviations from the null model are indicated by the red (busts) and blue (booms) rugs at the base of each panel. Time series of $\delta^{18}O$ values from the NGRIP ice core (d), $CO_2$ from the Dome C, Antarctica, ice core (e), and insolation seasonality (f) are provided for comparison. Data sources are in Methods.

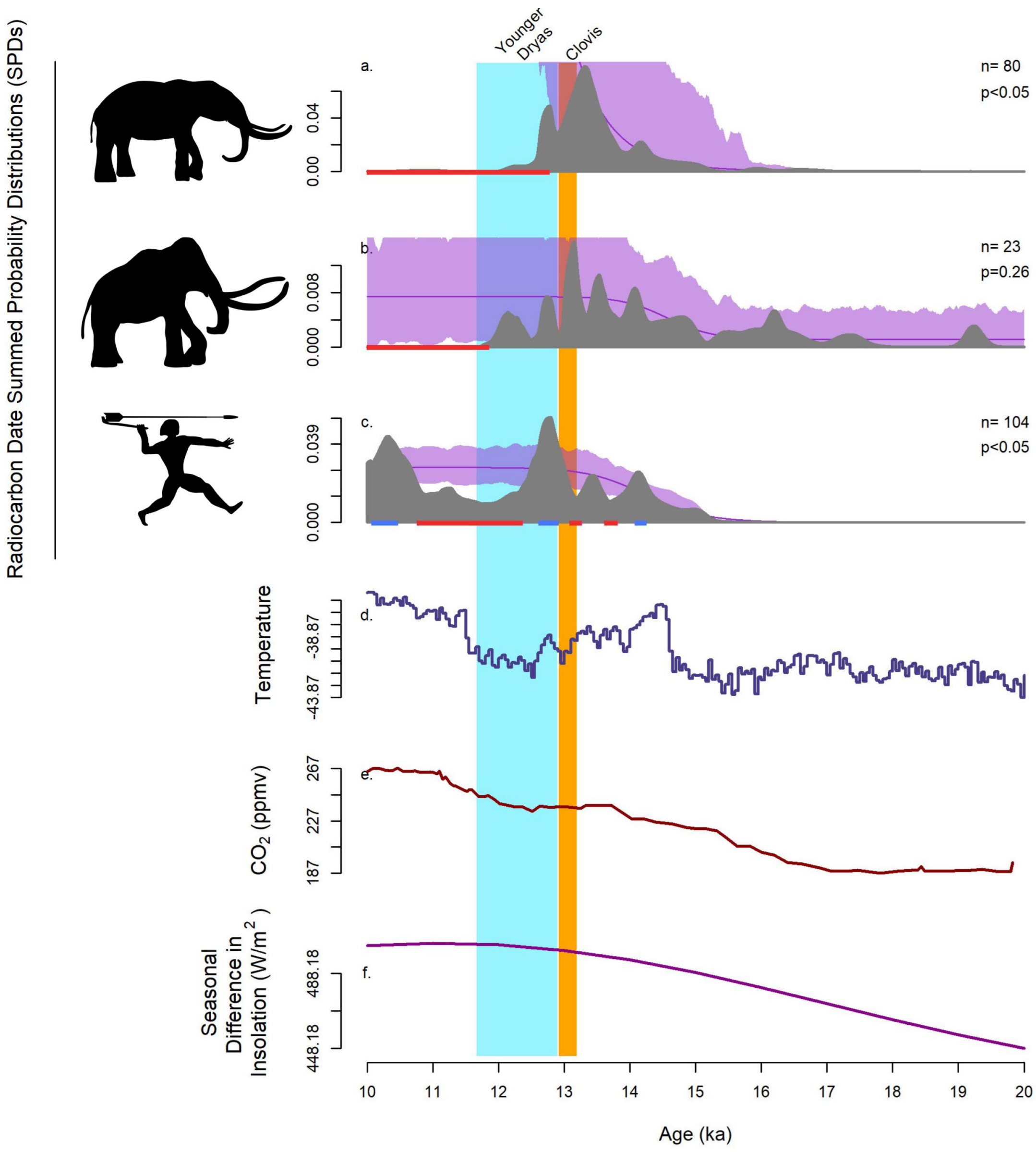


Supplementary Figure 8. SPDs for mastodon (a), mammoth (b), and human populations (c) through time for the Great Lakes region are indicated in gray. The null model of logistic growth is indicated by the purple line; the purple shading denotes the 95% confidence interval around the null model (see Methods). Statistically significant deviations from the null model are indicated by the red (busts) and blue (booms) rugs at the base of each panel. Time series of $\delta^{18}O$ values from the NGRIP ice core (d), $CO_2$ from the Dome C, Antarctica, ice core (e), and insolation seasonality (f) are provided for comparison. Data sources are in Methods.

**Supplementary References**

1. Halligan, J. J. *et al.* Pre-Clovis occupation 14,550 years ago at the Page-Ladson site, Florida, and the peopling of the Americas. *Sci Adv* **2016**, 2:e1600375 (2016).

2. Goldberg, P., Arpin, T. L. Micromorphological analysis of sediments from Meadowcroft Rockshelter, Pennsylvania: implications for radiocarbon dating. *J Field Arch* **26**, 325-342 (1999).

3. Jenkins, D. L. *et al.* Clovis age western stemmed projectile points and human coprolites at the Paisley Caves. *Science* **337**, 223-228 (2012).

4. Jenkins, D. L. *et al.* Geochronology, archaeological context, and DNA at the Paisley Caves. *Paleoamerican Odyssey*, eds Graf KE, Ketron CV, Waters MR (Texas A&M University Press, College Station, 2014) pp. 485-510.

5. Surovell, T. A, Pelton, S. R., Anderson-Sprecher, R., Myers, A. D. A test of Martin's overkill hypothesis using radiocarbon dates on extinct megafauna. *Proc Natl Acad Sci USA* **113** (4), 886-891 (2016).
