# Supplementary material for: Population reconstructions for humans and megafauna suggest mixed causes for North American Pleistocene extinctions
Source: Nat Commun. 2018 Dec 21;9:5441. doi: 10.1038/s41467-018-07897-1 (PMC6303330; doi:10.1038/s41467-018-07897-1)
Supplement: Supplementary file 3 — Description of Additional Supplementary Files [file 41467_2018_7897_MOESM3_ESM.docx]

Description of Additional Supplementary Information

Supplementary Data 1: Megafauna radiocarbon dates

Supplementary Data 2: R Code Used in This Analysis
