# Supplementary material for: Population reconstructions for humans and megafauna suggest mixed causes for North American Pleistocene extinctions
Source: Nat Commun. 2018 Dec 21;9:5441. doi: 10.1038/s41467-018-07897-1 (PMC6303330; doi:10.1038/s41467-018-07897-1)
Supplement: Supplementary file 5 — Supplementary Data 2 [file 41467_2018_7897_MOESM5_ESM.docx]

**###Broughton and Weitzel - R Code###**

#Load necessary packages ----

#install and load the 'rcarbon' package (available on GitHub) for constructing and analyzing summed probability distributions

library(devtools)

install_github("ahb108/rcarbon") #installs the most recent version of the package

library(rcarbon)

#load the 'segmented' package for segmented regression analysis

library(segmented)

#load the 'doParallel' package to allow the use of multiple cores when running the modelTest function from the 'rcarbon' package in order to speed up run time

library(doParallel)

#load the readxl package to read in Excel files

library(readxl)

#Load Data ----

##This script operates as if it were saved as an .R file in a folder containing several other folders: InputData, OutputTables, and Figures. All data to be loaded into the R workspace should be saved in the InputData folder and tables and figures produced by this script are saved in the OutputTables and Figures folders, respectively. This is evident in the file path names below##

#Set working directory

setwd("~/Research Projects/Current Research Projects/Megafauna and Human Population Modeling/Nature Submission/Less Old Stuff/020318")

#Load SI Dataset 1 (Megafauna radiocarbon dates)

USmegafauna <- read.csv("InputData/SIDataset1.csv") #.csv file of megafauna radiocarbon dates

#Load supplementary Bison dataset (compiled from CARD, Shapiro et al. 2004, and MacDonald 1981)

USbison <- read.csv("InputData/ FILENAME.csv")

#Load anthropogenic radiocarbon dates

##The Canadian Archaeological Radiocarbon Database (CARD) requests that their data not be reposted, so please visit http://www.canadianarchaeology.ca/ to locate and download relevant dates##

###For the vetted pre-Clovis supplementary analysis, replace all CARD dates prior to 13,200 cal BP (the beginning of the Clovis period) with the vetted pre-Clovis dates supplied in Table S2 (above)###

UShumdates <- read.csv("InputData/FILENAME.csv") #.csv file of anthropogenic radiocarbon dates from the CARD, cleaned following the protocols described in-text

UShumdates <- subset(UShumdates, Normalized.age<=12632) #subset CARD dates to eliminate allegedly anthropogenic dates from prior to 15000 cal BP (prior to generally accepted human colonization)

SW_HumanPop <- read.csv("InputData/ FILENAME.csv") #subset of CARD dates from the Southwest (see definition provided in-text)

SW_HumanPop <- subset(SW_HumanPop, Normalized_age<=12632) #subset CARD dates to eliminate allegedly anthropogenic dates from prior to 15000 cal BP (prior to generally accepted human colonization)

GL_HumanPop <- read.csv("InputData/ FILENAME.csv") #subset of CARD dates from the Great Lakes (see definition provided in-text)

GL_HumanPop <- subset(GL_HumanPop, Normalized<=12632) #subset CARD dates to eliminate allegedly anthropogenic dates from prior to 15000 cal BP (prior to generally accepted human colonization)

######################

#Prepare Climate Data#

######################

#CO2 Data

#Download .tab file from the following URL: https://doi.pangaea.de/10.1594/PANGAEA.472488

#Find the html file here: https://doi.pangaea.de/10.1594/PANGAEA.472488?format=html#download

#Download the .tab file and resave it as a .csv file under the same file name (using UTF-8 character encoding, as instructed by PANGAEA)

#Load .csv file

monnin.co2 <- read.csv("InputData/EDC_CO2_high_res.csv")

#Remove the first 16 rows (the metadata)

monnin.co2 <- monnin.co2[-c(1:17),]

#Give columns names

names(monnin.co2) <- c("Depth", "calBP", "CO2Meanppmv", "sigmameanppmv")

#Turn everything into numeric values instead of factors

monnin.co2$Depth <- as.numeric(levels(monnin.co2$Depth))[monnin.co2$Depth]

monnin.co2$calBP <- as.numeric(levels(monnin.co2$calBP))[monnin.co2$calBP]

monnin.co2$CO2Meanppmv <- as.numeric(levels(monnin.co2$CO2Meanppmv))[monnin.co2$CO2Meanppmv]

monnin.co2$sigmameanppmv <- as.numeric(levels(monnin.co2$sigmameanppmv))[monnin.co2$sigmameanppmv]

#Turn ka dates into cal BP by multiplying by 1000

monnin.co2$calBP <- monnin.co2$calBP*1000

#Write .csv

write.csv(monnin.co2, "InputData/Monnin.Co2Data.csv")

#NGRIP Data

#Download the .xls file here: http://www.nature.com.ezproxy.lib.uconn.edu/articles/nature02805#s1

NGRIP <- readxl::read_xls("InputData/nature02805-s1.xls", sheet=1)

NGRIP <- NGRIP[-c(1:28), ]

#Give columns names

names(NGRIP) <- c("BP", "D18O")

#Turn everything into numeric values instead of factors

NGRIP$BP <- as.numeric(as.character(NGRIP$BP))

NGRIP$D18O <- as.numeric(as.character(NGRIP$D18O))

#Turn supplied dates into cal BP by dividing by 1000 (the authors supplied dates with commas instead of periods, thus they're being interpreted incorrectly by R)

NGRIP$BP <- NGRIP$BP/1000

#Write .csv

write.csv(NGRIP, "InputData/NGRIP.csv")

#Load these now-prepared data

GRIP <- read.csv("InputData/NGRIP.csv") #Greenland ice core temperature data from NGRIP Project 2004

CO2 <- read.csv("InputData/Monnin.Co2Data.csv") #Carbon dioxide data from Monnin et al. 2001

#Load insolation seasonality data (already prepared)

Season <- read.csv("InputData/SeasonalInsolation.csv") #insolation data taken from Berger and Loutre 1991 (these data are supplied as they are calculates based on Berger and Loutre's data)

###########################################################################

#Calibrate using intcal13 curve (default) and create CalDates class object#

###########################################################################

#All megafauna and human radiocarbon dates are calibrated using the calibrate() function from the 'rcarbon' package. The Intcal13 curve is the default used in this function, and the function produces CalDates class objects which are necessary for constructing summed probability distributions (SPDs) using this package.

#Contiguous US ----

#US Mammoths

keepmam <- c("Mammuthus") #identify all Mammoth dates in this database

mamsub <- USmegafauna[USmegafauna$Taxon %in% keepmam, ] #keep only dates on mammoths for this analysis

USmamcal <- calibrate(mamsub$RCYBP, mamsub$Error) #calibrate dates

#US Mastodons

keepmast <- c("Mammut") #identify all Mastodon dates in this database

mastsub <- USmegafauna[USmegafauna$Taxon %in% keepmast, ] #keep only dates on Mastodons for this analysis

USmastcal <- calibrate(mastsub$RCYBP, mastsub$Error) #calibrate dates

#US Sloths

keepnoth <- c("Nothrotheriops") #identify all Nothrotheriops dates in this database

nothsub <- USmegafauna[USmegafauna$Taxon %in% keepnoth, ] #keep only dates on Nothrotheriops for this analysis

USnothcal <- calibrate(nothsub$RCYBP, nothsub$Error) #calibrate dates

#US Equus

keepeq <- c("Equus") #identify all Equus dates in this database

eqsub <- USmegafauna[USmegafauna$Taxon %in% keepeq, ] #keep only dates on Equus for this analysis

USeqcal <- calibrate(eqsub$RCYBP, eqsub$Error) #calibrate dates

#US Smilodon

keepsmil <- c("Smilodon") #identify all Smilodon dates in this database

smilsub <- USmegafauna[USmegafauna$Taxon %in% keepsmil, ] #keep only dates on Smilodon for this analysis

USsmilcal <- calibrate(smilsub$RCYBP, smilsub$Error) #calibrate dates

#US Humans

table(is.na(UShumdates$NA.Sigma)) #Two NA values exist in this vector

is.na(UShumdates$NA.Sigma) #Identify NA values in rows 499 and 59

UShumdates2 <- UShumdates[-c(59,499),] #remove rows with NA values

table(is.na(UShumdates2$NA.Sigma)) #No more NA values exist here

UShumcal <- calibrate(UShumdates2$Normalized.age, UShumdates2$NA.Sigma) #calibrate dates

#US Bison

#USbison <- UShumdates2[grep("bison", UShumdates2$Material.dated), ] #keep only CARD dates on Bison for this analysis

USbisoncal <- calibrate(USbison$Normalized.age, USbison$NA.Sigma) #calibrate dates

#Regional Analysis: Southwest ----

keepsw <- c("California", "Nevada", "Utah", "Arizona") #identify the states to include in the Southwest region: these states have some of the highest densities of megafauna dates in our database

SWmega <- USmegafauna[USmegafauna$State %in% keepsw, ] #keep only megafauna dates from this region

#SW Sloths

keepnoth <- c("Nothrotheriops") #identify the specific megafauna taxon to include in this taxon-specific analysis (Nothrotheriops is the most abundant taxon in this region)

SWnothsub <- SWmega[SWmega$Taxon %in% keepnoth, ] #keep only Nothrotheriops dates

SWnothcal <- calibrate(SWnothsub$RCYBP, SWnothsub$Error) #calibrate dates

#SW Mammoth

keepmam <- c("Mammuthus") #identify the specific megafauna taxon to include in this taxon-specific analysis (Mammuthus is the 2nd most abundant taxon in this region)

SWmamsub <- SWmega[SWmega$Taxon %in% keepmam, ] #keep only Mammoth dates

SWmamcal <- calibrate(SWmamsub$RCYBP, SWmamsub$Error) #calibrate dates

#SW Humans

SWhumcal <- calibrate(SW_HumanPop$Normalized_age, SW_HumanPop$NA_Sigma) #calibrate dates

#Regional Analysis: Great Lakes ----

keepGL <- c("Ontario", "New York", "Pennsylvania", "Ohio", "Indiana", "Illinois", "Michigan", "Wisconsin", "Minnesota") #identify the states and provinces to include in the Great Lakes region: these states have some of the highest densities of megafauna dates in our database

GLMega <- USmegafauna[USmegafauna$State %in% keepGL, ] #keep only megafauna dates from this region

#GL Mastodons

keepmast <- c("Mammut") #identify the specific megafauna taxon to include in this taxon-specific analysis (Mastodons are the most abundant taxon in this region)

GLmastsub <- GLMega[GLMega$Taxon %in% keepmast, ] #keep only Mastodon dates

GLmastcal <- calibrate(GLmastsub$RCYBP, GLmastsub$Error) #calibrate dates

#GL Mammoths

keepmam <- c("Mammuthus") #identify the specific megafauna taxon to include in this taxon-specific analysis (Mammoths are the 2nd most abundant taxon in this region)

GLmamsub <- GLMega[GLMega$Taxon %in% keepmam, ] #keep only Mammuthus dates

GLmamcal <- calibrate(GLmamsub$RCYBP, GLmamsub$Error) #calibrate dates

#GL Humans

GLhumcal <- calibrate(GL_HumanPop$Normalized, GL_HumanPop$NA_Sigma) #calibrate dates

################################################

#Create .csv of number of dates between 10-20ka#

################################################

USmamsamp <- mamsub[mamsub$RCYBP<=16774,]

USmamsamp <- USmamsamp[USmamsamp$RCYBP>=8838,]

USmastsamp <- mastsub[mastsub$RCYBP<=16774,]

USmastsamp <- USmastsamp[USmastsamp$RCYBP>=8838,]

USnothsamp <- nothsub[nothsub$RCYBP<=16774,]

USnothsamp <- USnothsamp[USnothsamp$RCYBP>=8838,]

USeqsamp <- eqsub[eqsub$RCYBP<=16774,]

USeqsamp <- USeqsamp[USeqsamp$RCYBP>=8838,]

USsmilsamp <- smilsub[smilsub$RCYBP<=16774,]

USsmilsamp <- USsmilsamp[USsmilsamp$RCYBP>=8838,]

UShumsamp <- UShumdates2[UShumdates2$Normalized.age<=12632,]

UShumsamp <- UShumsamp[UShumsamp$Normalized.age>=8838,]

USbisonsamp <- USbison[USbison$Normalized.age<=12632,]

USbisonsamp <- USbisonsamp[USbisonsamp$Normalized.age>=8838,]

SWnothsamp <- SWnothsub[SWnothsub$RCYBP<=16774,]

SWnothsamp <- SWnothsamp[SWnothsamp$RCYBP>=8838,]

SWmamsamp <- SWmamsub[SWmamsub$RCYBP<=16774,]

SWmamsamp <- SWmamsamp[SWmamsamp$RCYBP>=8838,]

SWhumsamp <- SW_HumanPop[SW_HumanPop$Normalized_age<=12632,]

SWhumsamp <- SWhumsamp[SWhumsamp$Normalized_age>=8838,]

GLmastsamp <- GLmastsub[GLmastsub$RCYBP<=16774,]

GLmastsamp <- GLmastsamp[GLmastsamp$RCYBP>=8838,]

GLmamsamp <- GLmamsub[GLmamsub$RCYBP<=16774,]

GLmamsamp <- GLmamsamp[GLmamsamp$RCYBP>=8838,]

GLhumsamp <- GL_HumanPop[GL_HumanPop$Normalized<=12632,]

GLhumsamp <- GLhumsamp[GLhumsamp$Normalized>=8838,]

datesamp <- data.frame("nUSmam"=nrow(USmamsamp), "nUSmast"=nrow(USmastsamp), "nUSnoth"=nrow(USnothsamp), 'nUSeq'=nrow(USeqsamp), 'nUSsmil'=nrow(USsmilsamp), "nUShum"=nrow(UShumsamp), "nUSbison"=nrow(USbisonsamp), "nSWnoth"=nrow(SWnothsamp), 'nSWmam'=nrow(SWmamsamp), "nSWhum"=nrow(SWhumsamp), "nGLmast"=nrow(GLmastsamp), 'nGLmam'=nrow(GLmamsamp), 'nGLhum'=nrow(GLhumsamp))

write.csv(datesamp, "OutputTables/RadiocarbonDateSampleSizes.csv")

################################################

#Create .csv of number of dates between 11-15ka#

################################################

USmamdens <- mamsub[mamsub$RCYBP<=12632,]

USmamdens <- USmamdens[USmamdens$RCYBP>=9627,]

USmastdens <- mastsub[mastsub$RCYBP<=12632,]

USmastdens <- USmastdens[USmastdens$RCYBP>=9627,]

USnothdens <- nothsub[nothsub$RCYBP<=12632,]

USnothdens <- USnothdens[USnothdens$RCYBP>=9627,]

USeqdens <- eqsub[eqsub$RCYBP<=12632,]

USotherdens <- USeqdens[USeqdens$RCYBP>=9627,]

USsmildens <- smilsub[smilsub$RCYBP<=12632,]

USsmildens <- USsmildens[USsmildens$RCYBP>=9627,]

UShumdens <- UShumdates2[UShumdates2$Normalized.age<=12632,]

UShumdens <- UShumdens[UShumdens$Normalized.age>=9627,]

USbisondens <- USbison[USbison$Normalized.age<=12632,]

USbisondens <- USbisondens[USbisondens$Normalized.age>=9627,]

SWnothdens <- SWnothsub[SWnothsub$RCYBP<=12632,]

SWnothdens <- SWnothdens[SWnothdens$RCYBP>=9627,]

SWmamdens <- SWmamsub[SWmamsub$RCYBP<=12632,]

SWmamdens <- SWmamdens[SWmamdens$RCYBP>=9627,]

SWhumdens <- SW_HumanPop[SW_HumanPop$Normalized_age<=12632,]

SWhumdens <- SWhumdens[SWhumdens$Normalized_age>=9627,]

GLmastdens <- GLmastsub[GLmastsub$RCYBP<=12632,]

GLmastdens <- GLmastdens[GLmastdens$RCYBP>=9627,]

GLmamdens <- GLmamsub[GLmamsub$RCYBP<=12632,]

GLmamdens <- GLmamdens[GLmamdens$RCYBP>=9627,]

GLhumdens <- GL_HumanPop[GL_HumanPop$Normalized<=12632,]

GLhumdens <- GLhumdens[GLhumdens$Normalized>=9627,]

datedens <- data.frame("USmam"=(nrow(USmamdens)/4000), "USmast"=(nrow(USmastdens)/4000), "USnoth"=(nrow(USnothdens)/4000), 'USeq'=(nrow(USeqdens)/4000), 'USsmil'=(nrow(USsmildens)/4000), "UShum"=(nrow(UShumdens)/4000), "USbison"=(nrow(USbisondens)/4000), "SWnoth"=(nrow(SWnothdens)/4000), "SWhum"=(nrow(SWhumdens)/4000), 'SWmam'=(nrow(SWmamdens)/4000), "GLmast"=(nrow(GLmastdens)/4000), 'GLmam'=(nrow(GLmamdens)/4000), "GLhum"=(nrow(GLhumdens)/4000))

write.csv(datedens, "OutputTables/RadiocarbonDateDensities.csv")

#############

#Create SPDs#

#############

#The CalDates class objects created above are used to construct summed probability distributions (SPDs).

#Contiguous US ----

#US Mammoths SPD

mamspd <- spd(USmamcal, timeRange=c(40000,8000), runm=200); plot(mamspd, xlim=c(8000,40000))

#US Mastodons SPD

mastspd <- spd(USmastcal, timeRange=c(40000,8000), runm=200); plot(mastspd, xlim=c(8000,40000))

#US Sloths SPD

nothspd <- spd(USnothcal, timeRange=c(40000,8000), runm=200); plot(nothspd, xlim=c(8000,40000))

#US Equus SPD

eqspd <- spd(USeqcal, timeRange=c(40000,8000), runm=200); plot(eqspd, xlim=c(8000,40000))

#US Smilodon SPD

smilspd <- spd(USsmilcal, timeRange=c(40000,8000), runm=200); plot(smilspd, xlim=c(8000,40000))

#US Human SPD

humspd <- spd(UShumcal, timeRange=c(40000,8000), runm=200); plot(humspd, xlim=c(8000,40000))

#US Bison SPD

bisonspd <- spd(USbisoncal, timeRange=c(40000,8000), runm=200); plot(bisonspd, xlim=c(8000,40000))

#Regional Analysis: Southwest ----

#SW Sloths SPD

SWnothspd <- spd(SWnothcal, timeRange=c(40000,8000), runm=200); plot(SWnothspd, xlim=c(8000,40000))

#SW Mammoth SPD

SWmamspd <- spd(SWmamcal, timeRange=c(40000,8000), runm=200); plot(SWmamspd, xlim=c(8000,40000))

#SW Human SPD

SWhumspd <- spd(SWhumcal, timeRange=c(40000,8000), runm=200); plot(SWhumspd, xlim=c(8000,40000))

#Regional Analysis: Great Lakes ----

#GL Mastodons SPD

GLmastspd <- spd(GLmastcal, timeRange=c(40000,8000), runm=200); plot(GLmastspd, xlim=c(8000,40000))

#GL Other Megafauna SPD

GLmamspd <- spd(GLmamcal, timeRange=c(40000,8000), runm=200); plot(GLmamspd, xlim=c(8000,40000))

#GL Human SPD

GLhumspd <- spd(GLhumcal, timeRange=c(40000,8000), runm=200); plot(GLhumspd, xlim=c(8000,40000))

###################################################

#Create data frame/.csv of SPD values through time#

###################################################

PopData <- data.frame("Date"=mamspd$grid$calBP, "USEq"=eqspd$grid$PrDens,"USSmil"=smilspd$grid$PrDens, "USMam"=mamspd$grid$PrDens, "USMast"=mastspd$grid$PrDens, "USNoth"=nothspd$grid$PrDens, "USHum"=humspd$grid$PrDens, "USBison"=bisonspd$grid$PrDens, "SWMam"=SWmamspd$grid$PrDens, "SWNoth"=SWnothspd$grid$PrDens, "SWHum"=SWhumspd$grid$PrDens, "GLMam"=GLmamspd$grid$PrDens, "GLMast"=GLmastspd$grid$PrDens, "GLHum"=GLhumspd$grid$PrDens)

write.csv(PopData, "OutputTables/PopData.csv")

#######################################

#Segmented regression analysis on SPDs#

#######################################

PopData <- read.csv("OutputTables/PopData.csv")

BptData <- PopData[c(25001:28301),] #subset data to model breakpoints only between 11700-15000: A narrower window of time surrounding the Clovis period and onset of the Younger Dryas helps to focus the function on the directional changes in the SPDs that are relevant to this analysis

#Using the 'segmented' package, segmented regression (or breakpoint) analysis optimizes the point at which to join pairs of generalized linear models in order to best describe the SPDs on which they are based. Segmented regression analysis therefore identifies the point at which the overall SPD changes direction between 11.7-15ka. This "breakpoint" corresponds to the date at which the population shifts from overall increasing to decreasing.

#US Breakpoints ----

#US Mammoths

BptUSMamSPD <- glm(USMam~Date, data=BptData, family=quasipoisson(link="log")); summary(BptUSMamSPD) #create a quasipoisson family GLM with log link

segUSmam <- segmented(BptUSMamSPD, seg.Z=~Date); summary(segUSmam) #locate the point at which to join paired GLMs

with(segUSmam, (null.deviance-deviance)/null.deviance) #Calculate a Pseudo R2 value

with(segUSmam, 1-pchisq(null.deviance-deviance , df.null-df.residual)) #Calculate a p value

#US Mastodons

BptUSMastSPD <- glm(USMast~Date, data=BptData, family=quasipoisson(link="log")); summary(BptUSMastSPD) #create a quasipoisson family GLM with log link

segUSmast <- segmented(BptUSMastSPD, seg.Z=~Date); summary(segUSmast) #locate the point at which to join paired GLMs

with(segUSmast, (null.deviance-deviance)/null.deviance) #Calculate a Pseudo R2 value

with(segUSmast, 1-pchisq(null.deviance-deviance , df.null-df.residual)) #Calculate a p value

#US Sloths

BptUSNothSPD <- glm(USNoth~Date, data=BptData, family=quasipoisson(link="log")); summary(BptUSNothSPD) #create a quasipoisson family GLM with log link

segUSnoth <- segmented(BptUSNothSPD, seg.Z=~Date); summary(segUSnoth) #locate the point at which to join paired GLMs

with(segUSnoth, (null.deviance-deviance)/null.deviance) #Calculate a Pseudo R2 value

with(segUSnoth, 1-pchisq(null.deviance-deviance , df.null-df.residual)) #Calculate a p value

#US Equus

BptUSEqSPD <- glm(USEq~Date, data=BptData, family=quasipoisson(link="log")); summary(BptUSEqSPD) #create a quasipoisson family GLM with log link

segUSeq <- segmented(BptUSEqSPD, seg.Z=~Date); summary(segUSeq) #locate the point at which to join paired GLMs

with(segUSeq, (null.deviance-deviance)/null.deviance) #Calculate a Pseudo R2 value

with(segUSeq, 1-pchisq(null.deviance-deviance , df.null-df.residual)) #Calculate a p value

#US Smilodon

BptUSSmilSPD <- glm(USSmil~Date, data=BptData, family=quasipoisson(link="log")); summary(BptUSSmilSPD) #create a quasipoisson family GLM with log link

segUSsmil <- segmented(BptUSSmilSPD, seg.Z=~Date); summary(segUSsmil) #locate the point at which to join paired GLMs

with(segUSsmil, (null.deviance-deviance)/null.deviance) #Calculate a Pseudo R2 value

with(segUSsmil, 1-pchisq(null.deviance-deviance , df.null-df.residual)) #Calculate a p value

#US Humans

BptUSHumSPD <- glm(USHum~Date, data=BptData, family=quasipoisson(link="log")); summary(BptUSHumSPD) #create a quasipoisson family GLM with log link

segUShum <- segmented(BptUSHumSPD, seg.Z=~Date); summary(segUShum) #locate the point at which to join paired GLMs

with(segUShum, (null.deviance-deviance)/null.deviance) #Calculate a Pseudo R2 value

with(segUShum, 1-pchisq(null.deviance-deviance , df.null-df.residual)) #Calculate a p value

#US Bison

BptUSBisonSPD <- glm(USBison~Date, data=BptData, family=quasipoisson(link="log")); summary(BptUSBisonSPD) #create a quasipoisson family GLM with log link

segUSbison <- segmented(BptUSBisonSPD, seg.Z=~Date); summary(segUSbison) #locate the point at which to join paired GLMs

with(segUSbison, (null.deviance-deviance)/null.deviance) #Calculate a Pseudo R2 value

with(segUSbison, 1-pchisq(null.deviance-deviance , df.null-df.residual)) #Calculate a p value

#Southwest Breakpoints ----

#SW Sloths

BptSWNothSPD <- glm(SWNoth~Date, data=BptData, family=quasipoisson(link="log")); summary(BptSWNothSPD) #create a quasipoisson family GLM with log link

segSWnoth <- segmented(BptSWNothSPD, seg.Z=~Date); summary(segSWnoth) #locate the point at which to join paired GLMs

with(segSWnoth, (null.deviance-deviance)/null.deviance) #Calculate a Pseudo R2 value

with(segSWnoth, 1-pchisq(null.deviance-deviance , df.null-df.residual)) #Calculate a p value

#SW Mammoths

BptSWMamSPD <- glm(SWMam~Date, data=BptData, family=quasipoisson(link="log")); summary(BptSWMamSPD) #create a quasipoisson family GLM with log link

segSWmam <- segmented(BptSWMamSPD, seg.Z=~Date); summary(segSWmam) #locate the point at which to join paired GLMs

with(segSWmam, (null.deviance-deviance)/null.deviance) #Calculate a Pseudo R2 value

with(segSWmam, 1-pchisq(null.deviance-deviance , df.null-df.residual)) #Calculate a p value

#SW Humans

BptSWHumSPD <- glm(SWHum~Date, data=BptData, family=quasipoisson(link="log")); summary(BptSWHumSPD) #create a quasipoisson family GLM with log link

segSWhum <- segmented(BptSWHumSPD, seg.Z=~Date); summary(segSWhum) #locate the point at which to join paired GLMs

with(segSWhum, (null.deviance-deviance)/null.deviance) #Calculate a Pseudo R2 value

with(segSWhum, 1-pchisq(null.deviance-deviance , df.null-df.residual)) #Calculate a p value

#Great Lakes Breakpoints ----

#GL Mastodons

BptGLMastSPD <- glm(GLMast~Date, data=BptData, family=quasipoisson(link="log")); summary(BptGLMastSPD) #create a quasipoisson family GLM with log link

segGLmast <- segmented(BptGLMastSPD, seg.Z=~Date); summary(segGLmast) #locate the point at which to join paired GLMs

with(segGLmast, (null.deviance-deviance)/null.deviance) #Calculate a Pseudo R2 value

with(segGLmast, 1-pchisq(null.deviance-deviance , df.null-df.residual)) #Calculate a p value

#GL Mammoths

BptGLMamSPD <- glm(GLMam~Date, data=BptData, family=quasipoisson(link="log")); summary(BptGLMamSPD) #create a quasipoisson family GLM with log link

segGLmam <- segmented(BptGLMamSPD, seg.Z=~Date); summary(segGLmam) #locate the point at which to join paired GLMs

with(segGLmam, (null.deviance-deviance)/null.deviance) #Calculate a Pseudo R2 value

with(segGLmam, 1-pchisq(null.deviance-deviance , df.null-df.residual)) #Calculate a p value

#GL Humans

BptGLHumSPD <- glm(GLHum~Date, data=BptData, family=quasipoisson(link="log")); summary(BptGLHumSPD) #create a quasipoisson family GLM with log link

segGLhum <- segmented(BptGLHumSPD, seg.Z=~Date); summary(segGLhum) #locate the point at which to join paired GLMs

with(segGLhum, (null.deviance-deviance)/null.deviance) #Calculate a Pseudo R2 value

with(segGLhum, 1-pchisq(null.deviance-deviance , df.null-df.residual)) #Calculate a p value

#Create data frame/.csv file with segmented regression results for each taxon ----

bptdf <- data.frame("Statistics"=c("Breakpoint","St.Err.","iterations"), "US Equus"=c(segUSeq$psi[2], segUSeq$psi[3], segUSeq$it), "US Smilodon"=c(segUSsmil$psi[2], segUSsmil$psi[3], segUSsmil$it), "US Mammoths"=c(segUSmam$psi[2], segUSmam$psi[3], segUSmam$it), "US Mastodons"=c(segUSmast$psi[2], segUSmast$psi[3], segUSmast$it), "US Sloths"=c(segUSnoth$psi[2], segUSnoth$psi[3], segUSnoth$it), "US Humans"=c(segUShum$psi[2], segUShum$psi[3], segUShum$it), "US Bison"=c(segUSbison$psi[2], segUSbison$psi[3], segUSbison$it), "SW Mammoths"=c(segSWmam$psi[2], segSWmam$psi[3], segSWmam$it), "SW Sloths"=c(segSWnoth$psi[2], segSWnoth$psi[3], segSWnoth$it), "SW Humans"=c(segSWhum$psi[2], segSWhum$psi[3], segSWhum$it), "GL Mammoths"=c(segGLmam$psi[2], segGLmam$psi[3], segGLmam$it), "GL Mastodons"=c(segGLmast$psi[2], segGLmast$psi[3], segGLmast$it), "GL Humans"=c(segGLhum$psi[2], segGLhum$psi[3], segGLhum$it))

write.csv(bptdf, "OutputTables/Breakpoints.csv")

###########################

#Compare SPD to Null Model#

###########################

#Compare the SPD for each taxon with a null model. Each null model is based on a GLM of each taxon relative to the date from 20ky to each taxon's specic breakpoint. These GLMs and null models therefore reflect the pre-breakpoint (and effectively pre-Younger Dryas) population trajectory for each species.

PopData <- read.csv("OutputTables/PopData.csv") #read in .csv of SPD values

memory.limit(size=25000) #manually set higher memory limit to handle large objects generated by modelTest function. Available memory is specific to computer specs.

#US Mammoths ----

USmamerr <- mamsub$Error #create vector of date error values for use with the modelTest function

USmamnull <- PopData[c(20001:(40000-segUSmam$psi[2])),] #limit SPD values to between 20ka and each taxon's specific breakpoint date

#create logistic null model

#5-parameter logistic growth model (Richard's Equation) using nplr package (3 and 4 parameter models are frequently unidentifiable when fitted to these data, but the nplr package will default to fewer parameters whenever possible)

USmamlogistic <- nplr::nplr(x=USmamnull$Date, y=USmamnull$USMam, LPweight=0) #fit model

plot(USmamlogistic, xlab="Date", ylab="Population", ylim=c(0,.05), xlim=c(4.00000,4.30103))

input <- log10(seq(10000, 20000, by=1)) #create range of dates across which to predict (the nplr function is log10 transforming the Date variable to aid in MLE, so feed it values on the log10 scale: 4.0 = 10,000, 4.30103 = 20,000)

USmamlogistic.pred <- USmamlogistic@pars$bottom + ((USmamlogistic@pars$top - USmamlogistic@pars$bottom)/((1 + (10^(USmamlogistic@pars$scal*(USmamlogistic@pars$xmid-input))))^USmamlogistic@pars$s)) #predict beyond breakpoint using Richard's Equation for a five-parameter logistic growth model (no predict function for nplr models)

lines(USmamlogistic.pred~input, col="blue")

plot(USMam~Date, data=USmamnull, xlim=c(10000,20000), type="l") #plot on true x scale

datevect <- 10^input #since the function log10 transformed "Date", transform it back

lines(USmamlogistic.pred~datevect, col="blue") #plot model fit

#create exponential null model

USmamglm <- glm(USMam~Date, data=USmamnull, family=quasipoisson); summary(USmamglm) #Use the range of SPD values obtained above to create a GLM of the relationship between population and time from 20ka to the taxon's breakpoint date. This effectively creates a model of pre-decline population growth.

plot(USMam~Date, data=USmamnull, xlab="", ylab="PrDens", pch=".", xlim=c(10000,20000), ylim=c(0,.25)) #plot the SPD values

newdata <- data.frame("Date"=seq(10000,20000, by=1)) #create date values for which to predict the GLM values

predUSmam <- predict(USmamglm, newdata=newdata, type="response", se=T) #predict the GLM values for the specified date range

lines(predUSmam$fit~newdata$Date, col="firebrick") #plot the predicted GLM values

nullglmUSmam <- data.frame("calBP"=newdata$Date, "PrDens"=predUSmam$fit) #add the GLM values and dates to a data frame that will be used to specify the null model to which to compare the SPD

USmammodel <- modelTest(USmamcal, errors=USmamerr, nsim=500, ncores=3, model="custom", predgrid=nullglmUSmam, timeRange=c(20000,10000), runm=200) #Use the modelTest function to compare the SPD to the specified null model (based on the pre-breakpoint GLM). Specify 500 simulations over the time range from 10-20ka. Additionally, with the 'doParallel' package loaded, specify the number of cores to use to run this function: this number will depend on the specs of the computer being used. Using multiple cores will drastically speed up the run time of this function.

plot(USmammodel) #plot the output of the modelTest function

USmammodel$pval #calculate the p value (regarding the difference between the SPD and null model)

#US Mastodons ----

USmasterr <- mastsub$Error

USmastnull <- PopData[c(20001:(40000-segUSmast$psi[2])),]

#create logistic null model

#5-parameter logistic growth model (Richard's Equation) using nplr package

USmastlogistic <- nplr::nplr(x=USmastnull$Date, y=USmastnull$USMast)

plot(USmastlogistic, xlab="Date", ylab="Population", ylim=c(0,.5), xlim=c(4.00000,4.30103))

input <- log10(seq(10000, 20000, by=1))

USmastlogistic.pred <- USmastlogistic@pars$bottom + ((USmastlogistic@pars$top - USmastlogistic@pars$bottom)/((1 + (10^(USmastlogistic@pars$scal*(USmastlogistic@pars$xmid-input))))^USmastlogistic@pars$s))

lines(USmastlogistic.pred~input, col="blue")

plot(USMast~Date, data=USmastnull, xlim=c(10000,20000), type="l", ylim=c(0,.4))

datevect <- 10^input

lines(USmastlogistic.pred~datevect, col="blue")

#create exponential null model

USmastglm <- glm(USMast~Date, data=USmastnull, family=quasipoisson); summary(USmastglm)

plot(USMast~Date, data=USmastnull, xlab="", ylab="PrDens", pch=".", xlim=c(10000,20000), ylim=c(0,.25))

newdata <- data.frame("Date"=seq(10000,20000, by=1))

predUSmast <- predict(USmastglm, newdata=newdata, type="response", se=T)

lines(predUSmast$fit~newdata$Date, col="firebrick")

nullglmUSmast <- data.frame("calBP"=newdata$Date, "PrDens"=predUSmast$fit)

USmastmodel <- modelTest(USmastcal, errors=USmasterr, nsim=500, ncores=3, model="custom", predgrid=nullglmUSmast, timeRange=c(20000,10000), runm=200)

plot(USmastmodel)

USmastmodel$pval

#US Sloths ----

USnotherr <- nothsub$Error

USnothnull <- PopData[c(20001:(40000-segUSnoth$psi[2])),]

#create logistic null model

#5-parameter logistic growth model (Richard's Equation) using nplr package

USnothlogistic <- nplr::nplr(x=USnothnull$Date, y=USnothnull$USNoth)

plot(USnothlogistic, xlab="Date", ylab="Population", ylim=c(0,.3), xlim=c(4.00000,4.30103))

input <- log10(seq(10000, 20000, by=1))

USnothlogistic.pred <- USnothlogistic@pars$bottom + ((USnothlogistic@pars$top - USnothlogistic@pars$bottom)/((1 + (10^(USnothlogistic@pars$scal*(USnothlogistic@pars$xmid-input))))^USnothlogistic@pars$s))

lines(USnothlogistic.pred~input, col="blue")

plot(USNoth~Date, data=USnothnull, xlim=c(10000,20000), type="l", ylim=c(0,.4))

datevect <- 10^input

lines(USnothlogistic.pred~datevect, col="blue")

#create exponential null model

USnothglm <- glm(USNoth~Date, data=USnothnull, family=quasipoisson); summary(USnothglm)

plot(USNoth~Date, data=USnothnull, xlab="", ylab="PrDens", pch=".", xlim=c(10000,20000), ylim=c(0,.25))

newdata <- data.frame("Date"=seq(10000,20000, by=1))

predUSnoth <- predict(USnothglm, newdata=newdata, type="response", se=T)

lines(predUSnoth$fit~newdata$Date, col="firebrick")

nullglmUSnoth <- data.frame("calBP"=newdata$Date, "PrDens"=predUSnoth$fit)

USnothmodel <- modelTest(USnothcal, errors=USnotherr, nsim=500, ncores=3, model="custom", predgrid=nullglmUSnoth, timeRange=c(20000,10000), runm=200)

plot(USnothmodel)

USnothmodel$pval

#US Equus ----

USeqerr <- eqsub$Error

USeqnull <- PopData[c(20001:(40000-segUSeq$psi[2])), ]

#create logistic null model

#5-parameter logistic growth model (Richard's Equation) using nplr package

USeqlogistic <- nplr::nplr(x=USeqnull$Date, y=USeqnull$USEq, npars=5)

plot(USeqlogistic, xlab="Date", ylab="Population", ylim=c(0,.1), xlim=c(4.00000,4.30103))

input <- log10(seq(10000, 20000, by=1))

USeqlogistic.pred <- USeqlogistic@pars$bottom + ((USeqlogistic@pars$top - USeqlogistic@pars$bottom)/((1 + (10^(USeqlogistic@pars$scal*(USeqlogistic@pars$xmid-input))))^USeqlogistic@pars$s))

lines(USeqlogistic.pred~input, col="blue")

plot(USEq~Date, data=USeqnull, xlim=c(10000,20000), type="l", ylim=c(0,.4))

datevect <- 10^input

lines(USeqlogistic.pred~datevect, col="blue") #this is a logistic model, but the top asymptote does not appear on this y-axis scale (the asymptote is around 5.0)

#create exponential null model

USeqglm <- glm(USEq~Date, data=USeqnull, family=quasipoisson); summary(USeqglm)

plot(USEq~Date, data=USeqnull, xlab="", ylab="PrDens", pch=".", xlim=c(10000,20000), ylim=c(0,.05))

newdata <- data.frame("Date"=seq(10000,20000, by=1))

predUSeq <- predict(USeqglm, newdata=newdata, type="response", se=T)

lines(predUSeq$fit~newdata$Date, col="firebrick")

nullglmUSeq <- data.frame("calBP"=newdata$Date, "PrDens"=predUSeq$fit)

USeqmodel <- modelTest(USeqcal, errors=USeqerr, nsim=500, ncores=3, model="custom", predgrid=nullglmUSeq, timeRange=c(20000,10000), runm=200)

plot(USeqmodel)

USeqmodel$pval

#US Smilodon ----

USsmilerr <- smilsub$Error

USsmilnull <- PopData[c(20001:(40000-segUSsmil$psi[2])), ]

#create logistic null model

#5-parameter logistic growth model (Richard's Equation) using nplr package

USsmillogistic <- nplr::nplr(x=USsmilnull$Date, y=USsmilnull$USSmil)

plot(USsmillogistic, xlab="Date", ylab="Population", ylim=c(0,.02), xlim=c(4.00000,4.30103))

input <- log10(seq(10000, 20000, by=1))

USsmillogistic.pred <- USsmillogistic@pars$bottom + ((USsmillogistic@pars$top - USsmillogistic@pars$bottom)/((1 + (10^(USsmillogistic@pars$scal*(USsmillogistic@pars$xmid-input))))^USsmillogistic@pars$s))

lines(USsmillogistic.pred~input, col="blue")

plot(USSmil~Date, data=USsmilnull, xlim=c(10000,20000), type="l", ylim=c(0,.02))

datevect <- 10^input

lines(USsmillogistic.pred~datevect, col="blue")

#create exponential null model

USsmilglm <- glm(USSmil~Date, data=USsmilnull, family=quasipoisson); summary(USsmilglm)

plot(USSmil~Date, data=USsmilnull, xlab="", ylab="PrDens", pch=".", xlim=c(10000,20000), ylim=c(0,.01))

newdata <- data.frame("Date"=seq(10000,20000, by=1))

predUSsmil <- predict(USsmilglm, newdata=newdata, type="response", se=T)

lines(predUSsmil$fit~newdata$Date, col="firebrick")

nullglmUSsmil <- data.frame("calBP"=newdata$Date, "PrDens"=predUSsmil$fit)

USsmilmodel <- modelTest(USsmilcal, errors=USsmilerr, nsim=500, ncores=3, model="custom", predgrid=nullglmUSsmil, timeRange=c(20000,10000), runm=200)

plot(USsmilmodel)

USsmilmodel$pval

#US Humans ----

UShumerr <- UShumdates2$NA.Sigma

UShumnull <- PopData[c(20001:(40000-segUShum$psi[2])),]

#create logistic null model

#5-parameter logistic growth model (Richard's Equation) using nplr package

UShumlogistic <- nplr::nplr(x=UShumnull$Date, y=UShumnull$USHum)

plot(UShumlogistic, xlab="Date", ylab="Population", ylim=c(0,.05), xlim=c(4.00000,4.30103)) #it appears that a logistic model cannot be fit well to these data

input <- log10(seq(10000, 20000, by=1))

UShumlogistic.pred <- UShumlogistic@pars$bottom + ((UShumlogistic@pars$top - UShumlogistic@pars$bottom)/((1 + (10^(UShumlogistic@pars$scal*(UShumlogistic@pars$xmid-input))))^UShumlogistic@pars$s))

lines(UShumlogistic.pred~input, col="blue")

plot(USHum~Date, data=UShumnull, xlim=c(10000,20000), type="l", ylim=c(0,.5))

datevect <- 10^input

lines(UShumlogistic.pred~datevect, col="blue")

#create exponential null model

UShumglm <- glm(USHum~Date, data=UShumnull, family=quasipoisson); summary(UShumglm)

plot(USHum~Date, data=UShumnull, xlab="", ylab="PrDens", pch=".", xlim=c(10000,20000), ylim=c(0,.03))

newdata <- data.frame("Date"=seq(10000,20000, by=1))

predUShum <- predict(UShumglm, newdata=newdata, type="response", se=T)

lines(predUShum$fit~newdata$Date, col="firebrick")

nullglmUShum <- data.frame("calBP"=newdata$Date, "PrDens"=predUShum$fit)

UShummodel <- modelTest(UShumcal, errors=UShumerr, nsim=500, ncores=3, model="custom", predgrid=nullglmUShum, timeRange=c(20000,10000), runm=200)

plot(UShummodel)

UShummodel$pval

#US Bison ----

USbisonerr <- USbison$NA.Sigma

USbisonnull <- PopData[c(20001:(40000-segUSbison$psi[2])),]

USbisonglm <- glm(USBison~Date, data=USbisonnull, family=quasipoisson); summary(USbisonglm)

plot(USBison~Date, data=USbisonnull, xlab="", ylab="PrDens", pch=".", xlim=c(10000,20000), ylim=c(0,.25))

newdata <- data.frame("Date"=seq(10000,20000, by=1))

predUSbison <- predict(USbisonglm, newdata=newdata, type="response", se=T)

lines(predUSbison$fit~newdata$Date, col="firebrick")

nullglmUSbison <- data.frame("calBP"=newdata$Date, "PrDens"=predUSbison$fit)

USbisonmodel <- modelTest(USbisoncal, errors=USbisonerr, nsim=500, ncores=3, model="custom", predgrid=nullglmUSbison, timeRange=c(20000,10000), runm=200)

plot(USbisonmodel)

USbisonmodel$pval

#SW Sloths ----

SWnotherr <- SWnothsub$Error

SWnothnull <- PopData[c(20001:(40000-segSWnoth$psi[2])), ]

#create logistic null model

#5-parameter logistic growth model (Richard's Equation) using nplr package

SWnothlogistic <- nplr::nplr(x=SWnothnull$Date, y=SWnothnull$SWNoth)

plot(SWnothlogistic, xlab="Date", ylab="Population", ylim=c(0,1), xlim=c(4.00000,4.30103))

input <- log10(seq(10000, 20000, by=1))

SWnothlogistic.pred <- SWnothlogistic@pars$bottom + ((SWnothlogistic@pars$top - SWnothlogistic@pars$bottom)/((1 + (10^(SWnothlogistic@pars$scal*(SWnothlogistic@pars$xmid-input))))^SWnothlogistic@pars$s))

lines(SWnothlogistic.pred~input, col="blue")

plot(SWNoth~Date, data=SWnothnull, xlim=c(10000,20000), type="l", ylim=c(0,.02))

datevect <- 10^input

lines(SWnothlogistic.pred~datevect, col="red")

#create exponential null model

SWnothglm <- glm(SWNoth~Date, data=SWnothnull, family=quasipoisson); summary(SWnothglm)

plot(SWNoth~Date, data=SWnothnull, xlab="", ylab="PrDens", pch=".", xlim=c(10000,20000), ylim=c(0,.25))

newdata <- data.frame("Date"=seq(10000,20000, by=1))

predSWnoth <- predict(SWnothglm, newdata=newdata, type="response", se=T)

lines(predSWnoth$fit~newdata$Date, col="firebrick")

nullglmSWnoth <- data.frame("calBP"=newdata$Date, "PrDens"=predSWnoth$fit)

SWnothmodel <- modelTest(SWnothcal, errors=SWnotherr, nsim=500, ncores=3, model="custom", predgrid=nullglmSWnoth, timeRange=c(20000,10000), runm=200)

plot(SWnothmodel)

SWnothmodel$pval

#SW Mammoth ----

SWmamerr <- SWmamsub$Error

SWmamnull <- PopData[c(20001:(40000-segSWmam$psi[2])), ]

#create logistic null model

#5-parameter logistic growth model (Richard's Equation) using nplr package

SWmamlogistic <- nplr::nplr(x=SWmamnull$Date, y=SWmamnull$SWMam)

plot(SWmamlogistic, xlab="Date", ylab="Population", ylim=c(0,.02), xlim=c(4.00000,4.30103))

input <- log10(seq(10000, 20000, by=1))

SWmamlogistic.pred <- SWmamlogistic@pars$bottom + ((SWmamlogistic@pars$top - SWmamlogistic@pars$bottom)/((1 + (10^(SWmamlogistic@pars$scal*(SWmamlogistic@pars$xmid-input))))^SWmamlogistic@pars$s))

lines(SWmamlogistic.pred~input, col="blue")

plot(SWMam~Date, data=SWmamnull, xlim=c(10000,20000), type="l", ylim=c(0,.02))

datevect <- 10^input

lines(SWmamlogistic.pred~datevect, col="red")

#create exponential null model

SWmamglm <- glm(SWMam~Date, data=SWmamnull, family=quasipoisson); summary(SWmamglm)

plot(SWMam~Date, data=SWmamnull, xlab="", ylab="PrDens", pch=".", xlim=c(10000,20000), ylim=c(0,.05))

newdata <- data.frame("Date"=seq(10000,20000, by=1))

predSWmam <- predict(SWmamglm, newdata=newdata, type="response", se=T)

lines(predSWmam$fit~newdata$Date, col="firebrick")

nullglmSWmam <- data.frame("calBP"=newdata$Date, "PrDens"=predSWmam$fit)

SWmammodel <- modelTest(SWmamcal, errors=SWmamerr, nsim=500, ncores=3, model="custom", predgrid=nullglmSWmam, timeRange=c(20000,10000), runm=200)

plot(SWmammodel)

SWmammodel$pval

#SW Humans ----

SWhumerr <- SW_HumanPop$NA_Sigma

SWhumnull <- PopData[c(20001:(40000-segSWhum$psi[2])),]

#create logistic null model

#5-parameter logistic growth model (Richard's Equation) using nplr package

SWhumlogistic <- nplr::nplr(x=SWhumnull$Date, y=SWhumnull$SWHum)

plot(SWhumlogistic, xlab="Date", ylab="Population", ylim=c(0,.5), xlim=c(4.00000,4.30103))

input <- log10(seq(10000, 20000, by=1))

SWhumlogistic.pred <- SWhumlogistic@pars$bottom + ((SWhumlogistic@pars$top - SWhumlogistic@pars$bottom)/((1 + (10^(SWhumlogistic@pars$scal*(SWhumlogistic@pars$xmid-input))))^SWhumlogistic@pars$s))

lines(SWhumlogistic.pred~input, col="blue")

plot(SWHum~Date, data=SWhumnull, xlim=c(10000,20000), type="l", ylim=c(0,.02))

datevect <- 10^input

lines(SWhumlogistic.pred~datevect, col="red")

#create exponential null model

SWhumglm <- glm(SWHum~Date, data=SWhumnull, family=quasipoisson); summary(SWhumglm)

plot(SWHum~Date, data=SWhumnull, xlab="", ylab="PrDens", pch=".", xlim=c(10000,20000), ylim=c(0,.5))

newdata <- data.frame("Date"=seq(10000,20000, by=1))

predSWhum <- predict(SWhumglm, newdata=newdata, type="response", se=T)

lines(predSWhum$fit~newdata$Date, col="firebrick")

nullglmSWhum <- data.frame("calBP"=newdata$Date, "PrDens"=predSWhum$fit)

SWhummodel <- modelTest(SWhumcal, errors=SWhumerr, nsim=500, ncores=3, model="custom", predgrid=nullglmSWhum, timeRange=c(20000,10000), runm=200)

plot(SWhummodel)

SWhummodel$pval

#GL Mastodons ----

GLmasterr <- GLmastsub$Error

GLmastnull <- PopData[c(20001:(40000-segGLmast$psi[2])), ]

#create logistic null model

#5-parameter logistic growth model (Richard's Equation) using nplr package

GLmastlogistic <- nplr::nplr(x=GLmastnull$Date, y=GLmastnull$GLMast)

plot(GLmastlogistic, xlab="Date", ylab="Population", ylim=c(0,.5), xlim=c(4.00000,4.30103))

input <- log10(seq(10000, 20000, by=1))

GLmastlogistic.pred <- GLmastlogistic@pars$bottom + ((GLmastlogistic@pars$top - GLmastlogistic@pars$bottom)/((1 + (10^(GLmastlogistic@pars$scal*(GLmastlogistic@pars$xmid-input))))^GLmastlogistic@pars$s))

lines(GLmastlogistic.pred~input, col="blue")

plot(GLMast~Date, data=GLmastnull, xlim=c(10000,20000), type="l", ylim=c(0,.2))

datevect <- 10^input

lines(GLmastlogistic.pred~datevect, col="red")

#create exponential null model

GLmastglm <- glm(GLMast~Date, data=GLmastnull, family=quasipoisson); summary(GLmastglm)

plot(GLMast~Date, data=GLmastnull, xlab="", ylab="PrDens", pch=".", xlim=c(10000,20000), ylim=c(0,.002))

newdata <- data.frame("Date"=seq(10000,20000, by=1))

predGLmast <- predict(GLmastglm, newdata=newdata, type="response", se=T)

lines(predGLmast$fit~newdata$Date, col="firebrick")

nullglmGLmast <- data.frame("calBP"=newdata$Date, "PrDens"=predGLmast$fit)

GLmastmodel <- modelTest(GLmastcal, errors=GLmasterr, nsim=500, ncores=3, model="custom", predgrid=nullglmGLmast, timeRange=c(20000,10000), runm=200)

plot(GLmastmodel)

GLmastmodel$pval

#GL Mammoth ----

GLmamerr <- GLmamsub$Error

GLmamnull <- PopData[c(20001:(40000-segGLmam$psi[2])), ]

#create logistic null model

#5-parameter logistic growth model (Richard's Equation) using nplr package

GLmamlogistic <- nplr::nplr(x=GLmamnull$Date, y=GLmamnull$GLMam)

plot(GLmamlogistic, xlab="Date", ylab="Population", ylim=c(0,.03), xlim=c(4.00000,4.30103))

input <- log10(seq(10000, 20000, by=1))

GLmamlogistic.pred <- GLmamlogistic@pars$bottom + ((GLmamlogistic@pars$top - GLmamlogistic@pars$bottom)/((1 + (10^(GLmamlogistic@pars$scal*(GLmamlogistic@pars$xmid-input))))^GLmamlogistic@pars$s))

lines(GLmamlogistic.pred~input, col="blue")

plot(GLMam~Date, data=GLmamnull, xlim=c(10000,20000), type="l", ylim=c(0,.02))

datevect <- 10^input

lines(GLmamlogistic.pred~datevect, col="red")

#create exponential null model

GLmamglm <- glm(GLMam~Date, data=GLmamnull, family=quasipoisson); summary(GLmamglm)

plot(GLMam~Date, data=GLmamnull, xlab="", ylab="PrDens", pch=".", xlim=c(10000,20000), ylim=c(0,.05))

newdata <- data.frame("Date"=seq(10000,20000, by=1))

predGLmam <- predict(GLmamglm, newdata=newdata, type="response", se=T)

lines(predGLmam$fit~newdata$Date, col="firebrick")

nullglmGLmam <- data.frame("calBP"=newdata$Date, "PrDens"=predGLmam$fit)

GLmammodel <- modelTest(GLmamcal, errors=GLmamerr, nsim=500, ncores=3, model="custom", predgrid=nullglmGLmam, timeRange=c(20000,10000), runm=200)

plot(GLmammodel)

GLmammodel$pval

#GL Humans ----

GLhumerr <- GL_HumanPop$NA_Sigma

GLhumnull <- PopData[c(20001:(40000-segGLhum$psi[2])),]

#create logistic null model

#5-parameter logistic growth model (Richard's Equation) using nplr package

GLhumlogistic <- nplr::nplr(x=GLhumnull$Date, y=GLhumnull$GLHum)

plot(GLhumlogistic, xlab="Date", ylab="Population", ylim=c(0,.04), xlim=c(4.00000,4.30103))

input <- log10(seq(10000, 20000, by=1))

GLhumlogistic.pred <- GLhumlogistic@pars$bottom + ((GLhumlogistic@pars$top - GLhumlogistic@pars$bottom)/((1 + (10^(GLhumlogistic@pars$scal*(GLhumlogistic@pars$xmid-input))))^GLhumlogistic@pars$s))

lines(GLhumlogistic.pred~input, col="blue")

plot(GLHum~Date, data=GLhumnull, xlim=c(10000,20000), type="l", ylim=c(0,.06))

datevect <- 10^input

lines(GLhumlogistic.pred~datevect, col="red")

#create exponential null model

GLhumglm <- glm(GLHum~Date, data=GLhumnull, family=quasipoisson); summary(GLhumglm)

plot(GLHum~Date, data=GLhumnull, xlab="", ylab="PrDens", pch=".", xlim=c(10000,20000), ylim=c(0,.5))

newdata <- data.frame("Date"=seq(10000,20000, by=1))

predGLhum <- predict(GLhumglm, newdata=newdata, type="response", se=T)

lines(predGLhum$fit~newdata$Date, col="firebrick")

nullglmGLhum <- data.frame("calBP"=newdata$Date, "PrDens"=predGLhum$fit)

GLhummodel <- modelTest(GLhumcal, errors=GLhumerr, nsim=500, ncores=3, model="custom", predgrid=nullglmGLhum, timeRange=c(20000,10000), runm=200)

plot(GLhummodel)

GLhummodel$pval

#Create data frame of SPDs and null models ----

#Contiguous United States

USmodeldf <- data.frame("Date"=rev(USeqmodel$fit$calBP), "EqNull"=rev(USeqmodel$fit$PrDens), "EqLo"=USeqmodel$result$lo, "EqHi"=USeqmodel$result$hi, "EqSPD"=USeqmodel$result$PrDens, "SmilNull"=rev(USsmilmodel$fit$PrDens), "SmilLo"=USsmilmodel$result$lo, "SmilHi"=USsmilmodel$result$hi, "SmilSPD"=USsmilmodel$result$PrDens, "MamNull"=rev(USmammodel$fit$PrDens), "MamLo"=USmammodel$result$lo, "MamHi"=USmammodel$result$hi, "MamSPD"=USmammodel$result$PrDens, "MastNull"=rev(USmastmodel$fit$PrDens), "MastLo"=USmastmodel$result$lo, "MastHi"=USmastmodel$result$hi, "MastSPD"=USmastmodel$result$PrDens, "NothNull"=rev(USnothmodel$fit$PrDens), "NothLo"=USnothmodel$result$lo, "NothHi"=USnothmodel$result$hi, "NothSPD"=USnothmodel$result$PrDens, "HumNull"=rev(UShummodel$fit$PrDens), "HumLo"=UShummodel$result$lo, "HumHi"=UShummodel$result$hi, "HumSPD"=UShummodel$result$PrDens, "BisonNull"=rev(USbisonmodel$fit$PrDens), "BisonLo"=USbisonmodel$result$lo, "BisonHi"=USbisonmodel$result$hi, "BisonSPD"=USbisonmodel$result$PrDens)

write.csv(USmodeldf, "OutputTables/SPDdataUSModels.csv")

#Southwest region

SWmodeldf <- data.frame("Date"=rev(SWmammodel$fit$calBP), "MamNull"=rev(SWmammodel$fit$PrDens), "MamLo"=SWmammodel$result$lo, "MamHi"=SWmammodel$result$hi, "MamSPD"=SWmammodel$result$PrDens, "NothNull"=rev(SWnothmodel$fit$PrDens), "NothLo"=SWnothmodel$result$lo, "NothHi"=SWnothmodel$result$hi, "NothSPD"=SWnothmodel$result$PrDens, "HumNull"=rev(SWhummodel$fit$PrDens), "HumLo"=SWhummodel$result$lo, "HumHi"=SWhummodel$result$hi, "HumSPD"=SWhummodel$result$PrDens)

write.csv(SWmodeldf, "OutputTables/SPDdataSWModels.csv")

#Great Lakes region

GLmodeldf <- data.frame("Date"=rev(GLmammodel$fit$calBP), "MamNull"=rev(GLmammodel$fit$PrDens), "MamLo"=GLmammodel$result$lo, "MamHi"=GLmammodel$result$hi, "MamSPD"=GLmammodel$result$PrDens, "MastNull"=rev(GLmastmodel$fit$PrDens), "MastLo"=GLmastmodel$result$lo, "MastHi"=GLmastmodel$result$hi, "MastSPD"=GLmastmodel$result$PrDens, "HumNull"=rev(GLhummodel$fit$PrDens), "HumLo"=GLhummodel$result$lo, "HumHi"=GLhummodel$result$hi, "HumSPD"=GLhummodel$result$PrDens)

write.csv(GLmodeldf, "OutputTables/SPDdataGLModels.csv")

###############################################################################

#Create vectors of significantly different SPD values (relative to null model)#

###############################################################################

#In order to show SPD deviations from the null models, we create a rug. This rug is then plotted at the bottom of each figure panel to highlight the dates for which the SPD values are significantly different (either higher or lower) than the null model.

#Create function to extract dates of periods of significant deviation from the null model ----

#This function will return the beginning and end dates of each period of significant deviation from the null model. It requires inputting a data frame of the structure of sigmegaloUS, sigmasthiGL, etc. which are created below by extracting the dates for which the SPD significantly deviates from the null model

minmaxrugdates <- function(hi, lo){

d_lo <- diff(lo$Date) #calculate the difference between each time step to identify sequences of significantly different dates for significantly lower values

d_hi <- diff(hi$Date)# do the same for the significantly greater values

rle_d_lo <- rle(d_lo) #calculate the length of these sequences

rle_d_hi <- rle(d_hi) #calculate the length of these sequences

lomax1 <- lo$Date[1] #pulls the first end date

lomin1 <- lo$Date[1+rle_d_lo$lengths[1]] #then the date for the beginning of that sequence

lomax2 <- lo$Date[1+rle_d_lo$lengths[1]+rle_d_lo$lengths[2]] #and so forth

lomin2 <- lo$Date[1+rle_d_lo$lengths[1]+rle_d_lo$lengths[2]+rle_d_lo$lengths[3]]

lomax3 <- lo$Date[1+rle_d_lo$lengths[1]+rle_d_lo$lengths[2]+rle_d_lo$lengths[3]+rle_d_lo$lengths[4]]

lomin3 <- lo$Date[1+rle_d_lo$lengths[1]+rle_d_lo$lengths[2]+rle_d_lo$lengths[3]+rle_d_lo$lengths[4]+rle_d_lo$lengths[5]]

lomax4 <- lo$Date[1+rle_d_lo$lengths[1]+rle_d_lo$lengths[2]+rle_d_lo$lengths[3]+rle_d_lo$lengths[4]+rle_d_lo$lengths[5]+rle_d_lo$lengths[6]]

lomin4 <- lo$Date[1+rle_d_lo$lengths[1]+rle_d_lo$lengths[2]+rle_d_lo$lengths[3]+rle_d_lo$lengths[4]+rle_d_lo$lengths[5]+rle_d_lo$lengths[6]+rle_d_lo$lengths[7]]

lomax5 <- lo$Date[1+rle_d_lo$lengths[1]+rle_d_lo$lengths[2]+rle_d_lo$lengths[3]+rle_d_lo$lengths[4]+rle_d_lo$lengths[5]+rle_d_lo$lengths[6]+rle_d_lo$lengths[7]+rle_d_lo$lengths[8]]

lomin5 <- lo$Date[1+rle_d_lo$lengths[1]+rle_d_lo$lengths[2]+rle_d_lo$lengths[3]+rle_d_lo$lengths[4]+rle_d_lo$lengths[5]+rle_d_lo$lengths[6]+rle_d_lo$lengths[7]+rle_d_lo$lengths[8]+rle_d_lo$lengths[9]]

lomax6 <- lo$Date[1+rle_d_lo$lengths[1]+rle_d_lo$lengths[2]+rle_d_lo$lengths[3]+rle_d_lo$lengths[4]+rle_d_lo$lengths[5]+rle_d_lo$lengths[6]+rle_d_lo$lengths[7]+rle_d_lo$lengths[8]+rle_d_lo$lengths[9]+rle_d_lo$lengths[10]]

lomin6 <- lo$Date[1+rle_d_lo$lengths[1]+rle_d_lo$lengths[2]+rle_d_lo$lengths[3]+rle_d_lo$lengths[4]+rle_d_lo$lengths[5]+rle_d_lo$lengths[6]+rle_d_lo$lengths[7]+rle_d_lo$lengths[8]+rle_d_lo$lengths[9]+rle_d_lo$lengths[10]+rle_d_lo$lengths[11]]

himax1 <- hi$Date[1] #pulls the first end date

himin1 <- hi$Date[1+rle_d_hi$lengths[1]] #then the date for the beginning of that sequence

himax2 <- hi$Date[1+rle_d_hi$lengths[1]+rle_d_hi$lengths[2]] #and so forth

himin2 <- hi$Date[1+rle_d_hi$lengths[1]+rle_d_hi$lengths[2]+rle_d_hi$lengths[3]]

himax3 <- hi$Date[1+rle_d_hi$lengths[1]+rle_d_hi$lengths[2]+rle_d_hi$lengths[3]+rle_d_hi$lengths[4]]

himin3 <- hi$Date[1+rle_d_hi$lengths[1]+rle_d_hi$lengths[2]+rle_d_hi$lengths[3]+rle_d_hi$lengths[4]+rle_d_hi$lengths[5]]

himax4 <- hi$Date[1+rle_d_hi$lengths[1]+rle_d_hi$lengths[2]+rle_d_hi$lengths[3]+rle_d_hi$lengths[4]+rle_d_hi$lengths[5]+rle_d_hi$lengths[6]]

himin4 <- hi$Date[1+rle_d_hi$lengths[1]+rle_d_hi$lengths[2]+rle_d_hi$lengths[3]+rle_d_hi$lengths[4]+rle_d_hi$lengths[5]+rle_d_hi$lengths[6]+rle_d_hi$lengths[7]]

himax5 <- hi$Date[1+rle_d_hi$lengths[1]+rle_d_hi$lengths[2]+rle_d_hi$lengths[3]+rle_d_hi$lengths[4]+rle_d_hi$lengths[5]+rle_d_hi$lengths[6]+rle_d_hi$lengths[7]+rle_d_hi$lengths[8]]

himin5 <- hi$Date[1+rle_d_hi$lengths[1]+rle_d_hi$lengths[2]+rle_d_hi$lengths[3]+rle_d_hi$lengths[4]+rle_d_hi$lengths[5]+rle_d_hi$lengths[6]+rle_d_hi$lengths[7]+rle_d_hi$lengths[8]+rle_d_hi$lengths[9]]

himax6 <- hi$Date[1+rle_d_hi$lengths[1]+rle_d_hi$lengths[2]+rle_d_hi$lengths[3]+rle_d_hi$lengths[4]+rle_d_hi$lengths[5]+rle_d_hi$lengths[6]+rle_d_hi$lengths[7]+rle_d_hi$lengths[8]+rle_d_hi$lengths[9]+rle_d_hi$lengths[10]]

himin6 <- hi$Date[1+rle_d_hi$lengths[1]+rle_d_hi$lengths[2]+rle_d_hi$lengths[3]+rle_d_hi$lengths[4]+rle_d_hi$lengths[5]+rle_d_hi$lengths[6]+rle_d_hi$lengths[7]+rle_d_hi$lengths[8]+rle_d_hi$lengths[9]+rle_d_hi$lengths[10]+rle_d_hi$lengths[11]]

minsmaxs <- data.frame(row.names=c("max1", "min1", "max2", "min2", "max3", "min3", "max4", "min4", "max5", "min5", "max6", "min6"), "Lo"=c(lomax1, lomin1, lomax2, lomin2, lomax3, lomin3, lomax4, lomin4, lomax5, lomin5, lomax6, lomin6), "Hi"=c(himax1, himin1, himax2, himin2, himax3, himin3, himax4, himin4, himax5, himin5, himax6, himin6))

print(minsmaxs)

}

#Contiguous US ----

USmodeldf <- read.csv("OutputTables/SPDdataUSModels.csv") #read in the SPD and null model data

sigeqloUS <- USmodeldf[USmodeldf$EqSPD<USmodeldf$EqLo,] #extract all dates for which the SPD value is less than the lower confidence inerval boundary for the null model

sigeqhiUS <- USmodeldf[USmodeldf$EqSPD>USmodeldf$EqHi,] #extract all dates for which the SPD value is greater than the higher confidence inerval boundary for the null model

USeqrug <- minmaxrugdates(hi=sigeqhiUS, lo=sigeqloUS) #use function created above to identify the beginnings and ends of each sequence of dates for which the SPD is significantly different from the null model.

write.csv(USeqrug, "OutputTables/RugTables/USEqRug.csv") #write this to a .csv

sigsmilloUS <- USmodeldf[USmodeldf$SmilSPD<USmodeldf$SmilLo,]

sigsmilhiUS <- USmodeldf[USmodeldf$SmilSPD>USmodeldf$SmilHi,]

USsmilrug <- minmaxrugdates(hi=sigsmilhiUS, lo=sigsmilloUS)

write.csv(USsmilrug, "OutputTables/RugTables/USSmilRug.csv")

sigmamloUS <- USmodeldf[USmodeldf$MamSPD<USmodeldf$MamLo,]

sigmamhiUS <- USmodeldf[USmodeldf$MamSPD>USmodeldf$MamHi,]

USmamrug <- minmaxrugdates(hi=sigmamhiUS, lo=sigmamloUS)

write.csv(USmamrug, "OutputTables/RugTables/USMamRug.csv")

sigmastloUS <- USmodeldf[USmodeldf$MastSPD<USmodeldf$MastLo,]

sigmasthiUS <- USmodeldf[USmodeldf$MastSPD>USmodeldf$MastHi,]

USmastrug <- minmaxrugdates(hi=sigmasthiUS, lo=sigmastloUS)

write.csv(USmastrug, "OutputTables/RugTables/USMastRug.csv")

signothloUS <- USmodeldf[USmodeldf$NothSPD<USmodeldf$NothLo,]

signothhiUS <- USmodeldf[USmodeldf$NothSPD>USmodeldf$NothHi,]

USnothrug <- minmaxrugdates(hi=signothhiUS, lo=signothloUS)

write.csv(USnothrug, "OutputTables/RugTables/USNothRug.csv")

sighumloUS <- USmodeldf[USmodeldf$HumSPD<USmodeldf$HumLo,]

sighumhiUS <- USmodeldf[USmodeldf$HumSPD>USmodeldf$HumHi,]

UShumrug <- minmaxrugdates(hi=sighumhiUS, lo=sighumloUS)

write.csv(UShumrug, "OutputTables/RugTables/USHumRug.csv")

sigbisonloUS <- USmodeldf[USmodeldf$BisonSPD<USmodeldf$BisonLo,]

sigbisonhiUS <- USmodeldf[USmodeldf$BisonSPD>USmodeldf$BisonHi,]

USbisonrug <- minmaxrugdates(hi=sigbisonhiUS, lo=sigbisonloUS)

write.csv(USbisonrug, "OutputTables/RugTables/USBisonRug.csv")

#Create rugs for US Equus

USeqruglo <- data.frame("Date"=sigeqloUS$Date, "SigEqLoSPD"=rep(0,nrow(sigeqloUS))) #create vector with values of 0 for all dates for which the SPD is lower than the low CI boundary (values of 0 will be plotted as a rug on the final figures)

USeqruglo <- USeqruglo[USeqruglo$Date<= 15000,] #remove dates prior to 15ka

USeqrughi <- data.frame("Date"=sigeqhiUS$Date, "SigEqHiSPD"=rep(0,nrow(sigeqhiUS))) #create a vector with values of 0 for all dates for which the SPD is greater than the upper CI boundary (values of 0 will be plotted as a rug on the final figures)

USeqrughi <- USeqrughi[USeqrughi$Date<= 15000,] #remove dates prior to 15ka

#Create rugs for US Smilodon

USsmilruglo <- data.frame("Date"=sigsmilloUS$Date, "SigSmilLoSPD"=rep(0,nrow(sigsmilloUS)))

USsmilruglo <- USsmilruglo[USsmilruglo$Date<= 15000,]

USsmilrughi <- data.frame("Date"=sigsmilhiUS$Date, "SigSmilHiSPD"=rep(0,nrow(sigsmilhiUS)))

USsmilrughi <- USsmilrughi[USsmilrughi$Date<= 15000,]

#Create rugs for US Mammoths

USmamruglo <- data.frame("Date"=sigmamloUS$Date, "SigMamLoSPD"=rep(0,nrow(sigmamloUS)))

USmamruglo <- USmamruglo[USmamruglo$Date<= 15000,]

USmamrughi <- data.frame("Date"=sigmamhiUS$Date, "SigMamHiSPD"=rep(0,nrow(sigmamhiUS)))

USmamrughi <- USmamrughi[USmamrughi$Date<= 15000,]

#Create rugs for US Mastodons

USmastruglo <- data.frame("Date"=sigmastloUS$Date, "SigMastLoSPD"=rep(0,nrow(sigmastloUS)))

USmastruglo <- USmastruglo[USmastruglo$Date<= 15000,]

USmastrughi <- data.frame("Date"=sigmasthiUS$Date, "SigMastHiSPD"=rep(0,nrow(sigmasthiUS)))

USmastrughi <- USmastrughi[USmastrughi$Date<= 15000,]

#Create rugs for US Sloths

USnothruglo <- data.frame("Date"=signothloUS$Date, "SigNothLoSPD"=rep(0,nrow(signothloUS)))

USnothruglo <- USnothruglo[USnothruglo$Date<= 15000,]

USnothrughi <- data.frame("Date"=signothhiUS$Date, "SigNothHiSPD"=rep(0,nrow(signothhiUS)))

USnothrughi <- USnothrughi[USnothrughi$Date<= 15000,]

#Create rugs for US Humans

UShumruglo <- data.frame("Date"=sighumloUS$Date, "SigHumLoSPD"=rep(0,nrow(sighumloUS)))

UShumruglo <- UShumruglo[UShumruglo$Date<= 15000,]

UShumrughi <- data.frame("Date"=sighumhiUS$Date, "SigHumHiSPD"=rep(0,nrow(sighumhiUS)))

UShumrughi <- UShumrughi[UShumrughi$Date<= 15000,]

#Create rugs for US Bison

USbisonruglo <- data.frame("Date"=sigbisonloUS$Date, "SigBisonLoSPD"=rep(0,nrow(sigbisonloUS)))

USbisonruglo <- USbisonruglo[USbisonruglo$Date<= 15000,]

USbisonrughi <- data.frame("Date"=sigbisonhiUS$Date, "SigBisonHiSPD"=rep(0,nrow(sigbisonhiUS)))

USbisonrughi <- USbisonrughi[USbisonrughi$Date<= 15000,]

#Regional Analysis: Southwest ----

SWmodeldf <- read.csv("OutputTables/SPDdataSWModels.csv") #read in the SPD and null model data

sigmamloSW <- SWmodeldf[SWmodeldf$MamSPD<SWmodeldf$MamLo,]

sigmamhiSW <- SWmodeldf[SWmodeldf$MamSPD>SWmodeldf$MamHi,]

SWmamrug <- minmaxrugdates(hi=sigmamhiSW, lo=sigmamloSW)

write.csv(SWmamrug, "OutputTables/RugTables/SWMamRug.csv")

signothloSW <- SWmodeldf[SWmodeldf$NothSPD<SWmodeldf$NothLo,]

signothhiSW <- SWmodeldf[SWmodeldf$NothSPD>SWmodeldf$NothHi,]

SWnothrug <- minmaxrugdates(hi=signothhiSW, lo=signothloSW)

write.csv(SWnothrug, "OutputTables/RugTables/SWNothRug.csv")

sighumloSW <- SWmodeldf[SWmodeldf$HumSPD<SWmodeldf$HumLo,]

sighumhiSW <- SWmodeldf[SWmodeldf$HumSPD>SWmodeldf$HumHi,]

SWhumrug <- minmaxrugdates(hi=sighumhiSW, lo=sighumloSW)

write.csv(SWhumrug, "OutputTables/RugTables/SWHumRug.csv")

#Create rugs for SW Mammoths

SWmamruglo <- data.frame("Date"=sigmamloSW$Date, "SigMamLoSPD"=rep(0,nrow(sigmamloSW))) #create vector with values of 0 for all dates for which the SPD is lower than the low CI boundary (values of 0 will be plotted as a rug on the final figures)

SWmamruglo <- SWmamruglo[SWmamruglo$Date<= 15000,] #remove dates prior to 15ka

SWmamrughi <- data.frame("Date"=sigmamhiSW$Date, "SigMamHiSPD"=rep(0,nrow(sigmamhiSW))) #create a vector with values of 0 for all dates for which the SPD is greater than the upper CI boundary (values of 0 will be plotted as a rug on the final figures)

SWmamrughi <- SWmamrughi[SWmamrughi$Date<= 15000,] #remove dates prior to 15ka

#Create rugs for SW Sloths

SWnothruglo <- data.frame("Date"=signothloSW$Date, "SigNothLoSPD"=rep(0,nrow(signothloSW)))

SWnothruglo <- SWnothruglo[SWnothruglo$Date<= 15000,]

SWnothrughi <- data.frame("Date"=signothhiSW$Date, "SigNothHiSPD"=rep(0,nrow(signothhiSW)))

SWnothrughi <- SWnothrughi[SWnothrughi$Date<= 15000,]

#Create rugs for SW Humans

SWhumruglo <- data.frame("Date"=sighumloSW$Date, "SigHumLoSPD"=rep(0,nrow(sighumloSW)))

SWhumruglo <- SWhumruglo[SWhumruglo$Date<= 15000,]

SWhumrughi <- data.frame("Date"=sighumhiSW$Date, "SigHumHiSPD"=rep(0,nrow(sighumhiSW)))

SWhumrughi <- SWhumrughi[SWhumrughi$Date<= 15000,]

#Regional Analysis: Great Lakes ----

GLmodeldf <- read.csv("OutputTables/SPDdataGLModels.csv") #read in the SPD and null model data

sigmamloGL <- GLmodeldf[GLmodeldf$MamSPD<GLmodeldf$MamLo,]

sigmamhiGL <- GLmodeldf[GLmodeldf$MamSPD>GLmodeldf$MamHi,]

GLmamrug <- minmaxrugdates(hi=sigmamhiGL, lo=sigmamloGL)

write.csv(GLmamrug, "OutputTables/RugTables/GLMamRug.csv")

sigmastloGL <- GLmodeldf[GLmodeldf$MastSPD<GLmodeldf$MastLo,]

sigmasthiGL <- GLmodeldf[GLmodeldf$MastSPD>GLmodeldf$MastHi,]

GLmastrug <- minmaxrugdates(hi=sigmasthiGL, lo=sigmastloGL)

write.csv(GLmastrug, "OutputTables/RugTables/GLMastRug.csv")

sighumloGL <- GLmodeldf[GLmodeldf$HumSPD<GLmodeldf$HumLo,]

sighumhiGL <- GLmodeldf[GLmodeldf$HumSPD>GLmodeldf$HumHi,]

GLhumrug <- minmaxrugdates(hi=sighumhiGL, lo=sighumloGL)

write.csv(GLhumrug, "OutputTables/RugTables/GLHumRug.csv")

#Create rugs for GL Mammoths

GLmamruglo <- data.frame("Date"=sigmamloGL$Date, "SigMamLoSPD"=rep(0,nrow(sigmamloGL))) #create vector with values of 0 for all dates for which the SPD is lower than the low CI boundary (values of 0 will be plotted as a rug on the final figures)

GLmamruglo <- GLmamruglo[GLmamruglo$Date<= 15000,] #remove dates prior to 15ka

GLmamrughi <- data.frame("Date"=sigmamhiGL$Date, "SigMamHiSPD"=rep(0,nrow(sigmamhiGL))) #create a vector with values of 0 for all dates for which the SPD is greater than the upper CI boundary (values of 0 will be plotted as a rug on the final figures)

GLmamrughi <- GLmamrughi[GLmamrughi$Date<= 15000,] #remove dates prior to 15ka

#Create rugs for GL Mastodons

GLmastruglo <- data.frame("Date"=sigmastloGL$Date, "SigMastLoSPD"=rep(0,nrow(sigmastloGL)))

GLmastruglo <- GLmastruglo[GLmastruglo$Date<= 15000,]

GLmastrughi <- data.frame("Date"=sigmasthiGL$Date, "SigMastHiSPD"=rep(0,nrow(sigmasthiGL)))

GLmastrughi <- GLmastrughi[GLmastrughi$Date<= 15000,]

#Create rugs for GL Humans

GLhumruglo <- data.frame("Date"=sighumloGL$Date, "SigHumLoSPD"=rep(0,nrow(sighumloGL)))

GLhumruglo <- GLhumruglo[GLhumruglo$Date<= 15000,]

GLhumrughi <- data.frame("Date"=sighumhiGL$Date, "SigHumHiSPD"=rep(0,nrow(sighumhiGL)))

GLhumrughi <- GLhumrughi[GLhumrughi$Date<= 15000,]

##################

#Plot SPD Figures#

##################

#Here, we plot all taxa which were analyzed on the same figure in addition to temperature, CO2, and insolation data published elsewhere.

GRIP2 <- GRIP[-c(1:399,802:4918),] #limit temperature data to 10-20ka

CO2.2 <- CO2[-c(1:102,177:183),] #limit CO2 data to 10-20ka

season2 <- Season[-c(1:10,22:1001),] #limit insolation data to 10-20ka

#read in .csv file of radiocarbon date sample sizes per taxon

datesamp <- read.csv("OutputTables/RadiocarbonDateSampleSizes.csv")

#Plot Contiguous US Results ----

jpeg("Figures/NAmObsSPD.jpeg", width = 8, height = 12, units = 'in', res = 300)

par(oma=c(6,6,0,0), mar=c(0,5,3,5) + 0.1, xpd=NA, mfrow=c(9,1))

plot(MamSPD~Date, data=USmodeldf, col="white", axes=F, xlab="", ylab="", xlim=c(10000,20000))

rect(11650, -0.38, 12900, max(USmodeldf$MamSPD)+.001, border=F, col="cadetblue1")

text(12600, .033, srt=45, pos=3, "Younger \nDryas")

rect(12900, -0.38, 13200, max(USmodeldf$MamSPD)+.001, border=F, col="orange")

text(13350,.033, srt=45, pos=3, "Clovis")

segments(7800, .035, 7800, -0.24)

text(10000, max(USmodeldf$MamSPD), "a.")

text(19200, max(USmodeldf$MamSPD), paste("n=", datesamp$nUSmam), pos=4)

text(19200, max(USmodeldf$MamSPD)-(max(USmodeldf$MamSPD/4)), paste("p<0.01"), pos=4)

par(xpd=F)

drkorch <- rgb(153, 50, 204, 127, maxColorValue=255)

polygon(c(rev(USmodeldf$Date), USmodeldf$Date), c(rev(USmodeldf$MamHi), USmodeldf$MamLo), col = drkorch, border = NA)

lines(USmodeldf$MamNull~USmodeldf$Date, col="darkorchid")

lines(MamSPD~Date, data=USmodeldf, type="h", col="gray50", lwd=2)

points(USmamruglo$SigMamLoSPD~USmamruglo$Date, pch="|", cex=.33, col="firebrick2")

points(USmamrughi$SigMamHiSPD~USmamrughi$Date, pch="|", cex=.33, col="royalblue1")

axis(2, line=0, at=seq(0,max(USmodeldf$MamSPD),round((max(USmodeldf$MamSPD)/4),3)), labels=T)

mtext(2, text="Mammoth", line=3.5, cex=.85)

plot(1, type="n", xlab="", ylab="", axes=F, xlim=c(10000, 20000), ylim=range(USmodeldf$MastSPD)); text(10000, max(USmodeldf$MastSPD)*.95, "b."); text(19200, max(USmodeldf$MastSPD)*.95, paste("n=", datesamp$nUSmast), pos=4); text(19200, (max(USmodeldf$MastSPD)*.95)-((max(USmodeldf$MastSPD)*.95)/4), paste("p<0.05"), pos=4); polygon(c(rev(USmodeldf$Date), USmodeldf$Date), c(rev(USmodeldf$MastHi), USmodeldf$MastLo), col = drkorch, border = NA); lines(USmodeldf$MastNull~USmodeldf$Date, col="darkorchid"); lines(MastSPD~Date, data=USmodeldf, type="h", col="gray50", lwd=2); points(USmastruglo$SigMastLoSPD~USmastruglo$Date, pch="|", cex=.33, col="firebrick2"); points(USmastrughi$SigMastHiSPD~USmastrughi$Date, pch="|", cex=.33, col="royalblue1"); axis(2, line=0, at=seq(0,max(USmodeldf$MastSPD),round((max(USmodeldf$MastSPD)/4),3)), labels=T); mtext(2, text="Mastodon", line=3.5, cex=.85)

plot(1, type="n", xlab="", ylab="", axes=F, xlim=c(10000, 20000), ylim=range(USmodeldf$NothSPD)); text(10000, max(USmodeldf$NothSPD)*.95, "c."); text(19200, max(USmodeldf$NothSPD)*.95, paste("n=", datesamp$nUSnoth), pos=4); text(19200, (max(USmodeldf$NothSPD)*.95)-((max(USmodeldf$NothSPD)*.95)/4), paste("p<0.01"), pos=4); polygon(c(rev(USmodeldf$Date), USmodeldf$Date), c(rev(USmodeldf$NothHi), USmodeldf$NothLo), col = drkorch, border = NA); lines(USmodeldf$NothNull~USmodeldf$Date, col="darkorchid"); lines(NothSPD~Date, data=USmodeldf, type="h", col="gray50", lwd=2); points(USnothruglo$SigNothLoSPD~USnothruglo$Date, pch="|", cex=.33, col="firebrick2"); points(USnothrughi$SigNothHiSPD~USnothrughi$Date, pch="|", cex=.33, col="royalblue1"); axis(2, line=0, at=seq(0,max(USmodeldf$NothSPD),round((max(USmodeldf$NothSPD)/4),3)), labels=T); mtext(2, text="Sloth", line=3.5, cex=.85)

mtext(2, text="Radiocarbon Date Summed Probability Distributions (SPDs)", line=8, cex=.85)

plot(1, type="n", xlab="", ylab="", axes=F, xlim=c(10000, 20000), ylim=range(USmodeldf$EqSPD)); text(10000, max(USmodeldf$EqSPD)*.95, "d."); text(19200, max(USmodeldf$EqSPD)*.95, paste("n=", datesamp$nUSeq), pos=4); text(19200, (max(USmodeldf$EqSPD)*.95)-((max(USmodeldf$EqSPD)*.95)/4), paste("p<0.05"), pos=4); polygon(c(rev(USmodeldf$Date), USmodeldf$Date), c(rev(USmodeldf$EqHi), USmodeldf$EqLo), col = drkorch, border = NA); lines(USmodeldf$EqNull~USmodeldf$Date, col="darkorchid"); lines(EqSPD~Date, data=USmodeldf, type="h", col="gray50", lwd=2); points(USeqruglo$SigEqLoSPD~USeqruglo$Date, pch="|", cex=.33, col="firebrick2"); points(USeqrughi$SigEqHiSPD~USeqrughi$Date, pch="|", cex=.33, col="royalblue1"); axis(2, line=0, at=seq(0,max(USmodeldf$EqSPD),round((max(USmodeldf$EqSPD)/4),3)), labels=T); mtext(2, text="Equus", line=3.2, cex=.85)

plot(1, type="n", xlab="", ylab="", axes=F, xlim=c(10000, 20000), ylim=range(USmodeldf$SmilSPD)); text(10000, max(USmodeldf$SmilSPD)*.95, "e."); text(19200, max(USmodeldf$SmilSPD)*.95, paste("n=", datesamp$nUSsmil), pos=4); text(19200, (max(USmodeldf$SmilSPD)*.95)-((max(USmodeldf$SmilSPD)*.95)/4), paste("p<0.01"), pos=4); polygon(c(rev(USmodeldf$Date), USmodeldf$Date), c(rev(USmodeldf$SmilHi), USmodeldf$SmilLo), col = drkorch, border = NA); lines(USmodeldf$SmilNull~USmodeldf$Date, col="darkorchid"); lines(SmilSPD~Date, data=USmodeldf, type="h", col="gray50", lwd=2); points(USsmilruglo$SigSmilLoSPD~USsmilruglo$Date, pch="|", cex=.33, col="firebrick2"); points(USsmilrughi$SigSmilHiSPD~USsmilrughi$Date, pch="|", cex=.33, col="royalblue1"); axis(2, line=0, at=seq(0,max(USmodeldf$SmilSPD),round((max(USmodeldf$SmilSPD)/4),3)), labels=T); mtext(2, text="Smilodon", line=3.2, cex=.85)

plot(1, type="n", xlab="", ylab="", axes=F, xlim=c(10000, 20000), ylim=range(USmodeldf$HumSPD)); text(10000, max(USmodeldf$HumSPD)*.95, "f."); text(19200, max(USmodeldf$HumSPD)*.95, paste("n=", datesamp$nUShum), pos=4); text(19200, (max(USmodeldf$HumSPD)*.95)-((max(USmodeldf$HumSPD)*.95)/4), paste("p<0.01"), pos=4); polygon(c(rev(USmodeldf$Date), USmodeldf$Date), c(rev(USmodeldf$HumHi), USmodeldf$HumLo), col = drkorch, border = NA); lines(USmodeldf$HumNull~USmodeldf$Date, col="darkorchid"); lines(HumSPD~Date, data=USmodeldf, type="h", col="gray50", lwd=2); points(UShumruglo$SigHumLoSPD~UShumruglo$Date, pch="|", cex=.33, col="firebrick2"); points(UShumrughi$SigHumHiSPD~UShumrughi$Date, pch="|", cex=.33, col="royalblue1"); axis(2, line=0, at=seq(0,max(USmodeldf$HumSPD),round((max(USmodeldf$HumSPD)/4),2)), labels=T); mtext(2, text="Human", line=3.5, cex=.85)

plot(1, type="n", xlab="", ylab="", axes=F, xlim=c(10000, 20000), ylim=range(GRIP2$D18O)); text(10000, max(GRIP2$D18O)*1.05, "g."); lines(GRIP2$D18O~GRIP2$BP, type="l", col="darkslateblue", lwd=2); axis(2, line=0, at=seq(min(GRIP2$D18O), max(GRIP2$D18O), 1), labels=T); mtext(2, text="Temperature", line=3.5, cex=.85)

plot(1, type="n", xlab="", ylab="", axes=F, xlim=c(10000, 20000), ylim=range(CO2.2$CO2Meanppmv)); text(10000, max(CO2.2$CO2Meanppmv)*.98, "h."); lines(CO2.2$CO2Meanppmv~CO2.2$calBP, type="l", col="darkred", lwd=2); axis(2, line=0, at=seq(min(CO2.2$CO2Meanppmv), max(CO2.2$CO2Meanppmv), 20), labels=T); mtext(2, text=expression("CO"[2]~"(ppmv)"), line=3.5, cex=.85)

plot(1, type="n", xlab="", ylab="", axes=F, xlim=c(10000, 20000), ylim=range(season2$Difference)); text(10000, max(season2$Difference)*.98, "i."); lines(season2$Difference~season2$BP, type="l", col="darkmagenta", lwd=2); axis(2, line=0, at=seq(min(season2$Difference), max(season2$Difference), 20), labels=T); mtext(2, text=expression(paste(" Seasonal \n Difference in \n Insolation (W/m"^"2"~")")), line=3.5, cex=.85)

axis(1, line=0.5, at=seq(10000,20000, by=1000), labels=c(seq(10,20,1)))

mtext(1, text="Age (ka)", line=3.75, cex=.8)

dev.off()

#Plot Southwestern Results ----

jpeg("Figures/SWObsSPD.jpeg", width = 7, height = 8, units = 'in', res = 300)

par(oma=c(6,6,0,0), mar=c(0,5,3,5) + 0.1, xpd=NA, mfrow=c(6,1))

plot(NothSPD~Date, data=SWmodeldf, col="white", axes=F, xlab="", ylab="", xlim=c(10000,20000))

rect(11650, -.23, 12900, max(SWmodeldf$NothSPD)+.001, border=F, col="cadetblue1")

text(12600, .03, srt=45, pos=3, "Younger \nDryas")

rect(12900, -.23, 13200, max(SWmodeldf$NothSPD)+.001, border=F, col="orange")

text(13350,.03, srt=45, pos=3, "Clovis")

text(10000, max(SWmodeldf$NothSPD), "a.")

text(19200, max(SWmodeldf$NothSPD), paste("n=", datesamp$nSWnoth), pos=4)

text(19200, max(SWmodeldf$NothSPD)-(max(SWmodeldf$NothSPD/4)), paste("p<0.01"), pos=4)

segments(7700,.027,7700,-.1)

par(xpd=F)

drkorch <- rgb(153, 50, 204, 127, maxColorValue=255)

polygon(c(rev(SWmodeldf$Date), SWmodeldf$Date), c(rev(SWmodeldf$NothHi), SWmodeldf$NothLo), col = drkorch, border = NA)

lines(SWmodeldf$NothNull~SWmodeldf$Date, col="darkorchid")

lines(NothSPD~Date, data=SWmodeldf, type="h", col="gray50", lwd=2)

points(SWnothruglo$SigNothLoSPD~SWnothruglo$Date, pch="|", cex=.33, col="firebrick2")

points(SWnothrughi$SigNothHiSPD~SWnothrughi$Date, pch="|", cex=.33, col="royalblue1")

axis(2, line=0, at=seq(0,max(SWmodeldf$NothSPD),round((max(SWmodeldf$NothSPD)/4),3)), labels=T)

mtext(2, text="Sloth", line=3.5, cex=.85)

plot(1, type="n", xlab="", ylab="", axes=F, xlim=c(10000, 20000), ylim=range(SWmodeldf$MamSPD)); text(10000, max(SWmodeldf$MamSPD)*.95, "b."); text(19200, max(SWmodeldf$MamSPD)*.95, paste("n=", datesamp$nSWmam), pos=4); text(19200, (max(SWmodeldf$MamSPD)*.95)-((max(SWmodeldf$MamSPD)*.95)/4), paste("p<0.01"), pos=4); polygon(c(rev(SWmodeldf$Date), SWmodeldf$Date), c(rev(SWmodeldf$MamHi), SWmodeldf$MamLo), col = drkorch, border = NA); lines(SWmodeldf$MamNull~SWmodeldf$Date, col="darkorchid"); lines(MamSPD~Date, data=SWmodeldf, type="h", col="gray50", lwd=2); points(SWmamruglo$SigMamLoSPD~SWmamruglo$Date, pch="|", cex=.33, col="firebrick2"); points(SWmamrughi$SigMamHiSPD~SWmamrughi$Date, pch="|", cex=.33, col="royalblue1"); axis(2, line=0, at=seq(0, max(SWmodeldf$MamSPD), round((max(SWmodeldf$MamSPD)/4), 3)), labels=T); mtext(2, text="Mammoth", line=3.2, cex=.85)

mtext(2, text="Radiocarbon Date Summed Probability Distributions (SPDs)", line=8, cex=.85)

plot(1, type="n", xlab="", ylab="", axes=F, xlim=c(10000, 20000), ylim=range(SWmodeldf$HumSPD)); text(10000, max(SWmodeldf$HumSPD)*.95, "c."); text(19200, max(SWmodeldf$HumSPD)*.95, paste("n=", datesamp$nSWhum), pos=4); text(19200, (max(SWmodeldf$HumSPD)*.95)-((max(SWmodeldf$HumSPD)*.95)/4), paste("p<0.01"), pos=4); polygon(c(rev(SWmodeldf$Date), SWmodeldf$Date), c(rev(SWmodeldf$HumHi), SWmodeldf$HumLo), col = drkorch, border = NA); lines(SWmodeldf$HumNull~SWmodeldf$Date, col="darkorchid"); lines(HumSPD~Date, data=SWmodeldf, type="h", col="gray50", lwd=2); points(SWhumruglo$SigHumLoSPD~SWhumruglo$Date, pch="|", cex=.33, col="firebrick2"); axis(2, line=0, at=seq(0, max(SWmodeldf$HumSPD), round((max(SWmodeldf$HumSPD)/4), 3)), labels=T); mtext(2, text="Human", line=3.5, cex=.85)

plot(1, type="n", xlab="", ylab="", axes=F, xlim=c(10000, 20000), ylim=range(GRIP2$D18O)); text(10000, max(GRIP2$D18O)*1.05, "d."); lines(GRIP2$D18O~GRIP2$BP, type="l", col="darkslateblue", lwd=2); axis(2, line=0, at=seq(min(GRIP2$D18O), max(GRIP2$D18O), 1), labels=T); mtext(2, text="Temperature", line=3.5, cex=.85)

plot(1, type="n", xlab="", ylab="", axes=F, xlim=c(10000, 20000), ylim=range(CO2.2$CO2Meanppmv)); text(10000, max(CO2.2$CO2Meanppmv)*.98, "e."); lines(CO2.2$CO2Meanppmv~CO2.2$calBP, type="l", col="darkred", lwd=2); axis(2, line=0, at=seq(min(CO2.2$CO2Meanppmv), max(CO2.2$CO2Meanppmv), 20), labels=T); mtext(2, text=expression("CO"[2]~"(ppmv)"), line=3.5, cex=.85)

plot(1, type="n", xlab="", ylab="", axes=F, xlim=c(10000, 20000), ylim=range(season2$Difference)); text(10000, max(season2$Difference)*.97, "f."); lines(season2$Difference~season2$BP, type="l", col="darkmagenta", lwd=2); axis(2, line=0, at=seq(min(season2$Difference), max(season2$Difference), 20), labels=T); mtext(2, text=expression(paste(" Seasonal \n Difference in \n Insolation (W/m"^"2"~")")), line=3.5, cex=.85)

axis(1, line=0.5, at=seq(10000,20000, by=1000), labels=c(seq(10,20,1)))

mtext(1, text="Age (ka)", line=3.75, cex=.8)

dev.off()

#Plot Great Lakes Results ----

jpeg("Figures/GLObsSPD.jpeg", width = 7, height = 8, units = 'in', res = 300)

par(oma=c(6,6,0,0), mar=c(0,5,3,5) + 0.1, xpd=NA, mfrow=c(6,1))

plot(MastSPD~Date, data=GLmodeldf, col="white", axes=F, xlab="", ylab="", xlim=c(10000,20000))

rect(11650, -.66, 12900, max(GLmodeldf$MastSPD)+.0032, border=F, col="cadetblue1")

text(12600, .086, srt=45, pos=3, "Younger \nDryas")

rect(12900, -.66, 13200, max(GLmodeldf$MastSPD)+.0032, border=F, col="orange")

text(13350,.086, srt=45, pos=3, "Clovis")

text(10000, max(GLmodeldf$MastSPD), "a.")

text(19200, max(GLmodeldf$MastSPD), paste("n=", datesamp$nGLmast), pos=4)

text(19200, max(GLmodeldf$MastSPD)-(max(GLmodeldf$MastSPD/4)), paste("p<0.01"), pos=4)

segments(7700, .086, 7700, -.285)

par(xpd=F)

drkorch <- rgb(153, 50, 204, 127, maxColorValue=255)

polygon(c(rev(GLmodeldf$Date), GLmodeldf$Date), c(rev(GLmodeldf$MastHi), GLmodeldf$MastLo), col = drkorch, border = NA)

lines(GLmodeldf$MastNull~GLmodeldf$Date, col="darkorchid")

lines(MastSPD~Date, data=GLmodeldf, type="h", col="gray50", lwd=2)

points(GLmastruglo$SigMastLoSPD~GLmastruglo$Date, pch="|", cex=.33, col="firebrick2")

points(GLmastrughi$SigMastHiSPD~GLmastrughi$Date, pch="|", cex=.33, col="royalblue1")

axis(2, line=0, at=seq(0,max(GLmodeldf$MastSPD),round((max(GLmodeldf$MastSPD)/4),3)), labels=T)

mtext(2, text="Mastodon", line=3.5, cex=.85)

plot(1, type="n", xlab="", ylab="", axes=F, xlim=c(10000, 20000), ylim=range(GLmodeldf$MamSPD)); text(10000, max(GLmodeldf$MamSPD)*.95, "b."); text(19200, max(GLmodeldf$MamSPD)*.95, paste("n=", datesamp$nGLmam), pos=4); text(19200, (max(GLmodeldf$MamSPD)*.95)-((max(GLmodeldf$MamSPD)*.95)/4), paste("p<0.05"), pos=4); polygon(c(rev(GLmodeldf$Date), GLmodeldf$Date), c(rev(GLmodeldf$MamHi), GLmodeldf$MamLo), col = drkorch, border = NA); lines(GLmodeldf$MamNull~GLmodeldf$Date, col="darkorchid"); lines(MamSPD~Date, data=GLmodeldf, type="h", col="gray50", lwd=2); points(GLmamruglo$SigMamLoSPD~GLmamruglo$Date, pch="|", cex=.33, col="firebrick2"); points(GLmamrughi$SigMamHiSPD~GLmamrughi$Date, pch="|", cex=.33, col="royalblue1"); axis(2, line=0, at=seq(0, max(GLmodeldf$MamSPD), round((max(GLmodeldf$MamSPD)/4), 3)), labels=T); mtext(2, text="Mammoth", line=3.2, cex=.85)

mtext(2, text="Radiocarbon Date Summed Probability Distributions (SPDs)", line=8, cex=.85)

plot(1, type="n", xlab="", ylab="", axes=F, xlim=c(10000, 20000), ylim=range(GLmodeldf$HumSPD)); text(10000, max(GLmodeldf$HumSPD)*.95, "c."); text(19200, max(GLmodeldf$HumSPD)*.95, paste("n=", datesamp$nGLhum), pos=4); text(19200, (max(GLmodeldf$HumSPD)*.95)-((max(GLmodeldf$HumSPD)*.95)/4), paste("p<0.01"), pos=4); polygon(c(rev(GLmodeldf$Date), GLmodeldf$Date), c(rev(GLmodeldf$HumHi), GLmodeldf$HumLo), col = drkorch, border = NA); lines(GLmodeldf$HumNull~GLmodeldf$Date, col="darkorchid"); lines(HumSPD~Date, data=GLmodeldf, type="h", col="gray50", lwd=2); points(GLhumruglo$SigHumLoSPD~GLhumruglo$Date, pch="|", cex=.33, col="firebrick2"); points(GLhumrughi$SigHumHiSPD~GLhumrughi$Date, pch="|", cex=.33, col="royalblue1"); axis(2, line=0, at=seq(0, max(GLmodeldf$HumSPD), round((max(GLmodeldf$HumSPD)/4), 3)), labels=T); mtext(2, text="Human", line=3.5, cex=.85)

plot(1, type="n", xlab="", ylab="", axes=F, xlim=c(10000, 20000), ylim=range(GRIP2$D18O)); text(10000, max(GRIP2$D18O)*1.05, "d."); lines(GRIP2$D18O~GRIP2$BP, type="l", col="darkslateblue", lwd=2); axis(2, line=0, at=seq(min(GRIP2$D18O), max(GRIP2$D18O), 1), labels=T); mtext(2, text="Temperature", line=3.5, cex=.85)

plot(1, type="n", xlab="", ylab="", axes=F, xlim=c(10000, 20000), ylim=range(CO2.2$CO2Meanppmv)); text(10000, max(CO2.2$CO2Meanppmv)*.98, "e."); lines(CO2.2$CO2Meanppmv~CO2.2$calBP, type="l", col="darkred", lwd=2); axis(2, line=0, at=seq(min(CO2.2$CO2Meanppmv), max(CO2.2$CO2Meanppmv), 20), labels=T); mtext(2, text=expression("CO"[2]~"(ppmv)"), line=3.5, cex=.85)

plot(1, type="n", xlab="", ylab="", axes=F, xlim=c(10000, 20000), ylim=range(season2$Difference)); text(10000, max(season2$Difference)*.97, "f."); lines(season2$Difference~season2$BP, type="l", col="darkmagenta", lwd=2); axis(2, line=0, at=seq(min(season2$Difference), max(season2$Difference), 20), labels=T); mtext(2, text=expression(paste(" Seasonal \n Difference in \n Insolation (W/m"^"2"~")")), line=3.5, cex=.85)

axis(1, line=0.5, at=seq(10000,20000, by=1000), labels=c(seq(10,20,1)))

mtext(1, text="Age (ka)", line=3.75, cex=.8)

dev.off()

#Plot Bison results ----

jpeg("Figures/BisonObsSPD.jpeg", width = 7, height = 7, units = 'in', res = 300)

par(oma=c(6,6,0,0), mar=c(0,5,3,5) + 0.1, xpd=NA, mfrow=c(5,1))

plot(BisonSPD~Date, data=USmodeldf, col="white", axes=F, xlab="", ylab="", xlim=c(10000,20000))

rect(11650, -.31, 12900, max(USmodeldf$BisonSPD)+.0032, border=F, col="cadetblue1")

text(12600, .053, srt=45, pos=3, "Younger \nDryas")

rect(12900, -.31, 13200, max(USmodeldf$BisonSPD)+.0032, border=F, col="orange")

text(13350,.053, srt=45, pos=3, "Clovis")

text(10000, max(USmodeldf$BisonSPD), "a.")

text(19200, max(USmodeldf$BisonSPD), paste("n=", datesamp$nUSbison), pos=4)

text(19200, max(USmodeldf$BisonSPD)-(max(USmodeldf$BisonSPD/4)), paste("p<0.01"), pos=4)

segments(7700, .053, 7700, -.082)

par(xpd=F)

drkorch <- rgb(153, 50, 204, 127, maxColorValue=255)

polygon(c(rev(USmodeldf$Date), USmodeldf$Date), c(rev(USmodeldf$BisonHi), USmodeldf$BisonLo), col = drkorch, border = NA)

lines(USmodeldf$BisonNull~USmodeldf$Date, col="darkorchid")

lines(BisonSPD~Date, data=USmodeldf, type="h", col="gray50", lwd=2)

points(USbisonruglo$SigBisonLoSPD~USbisonruglo$Date, pch="|", cex=.33, col="firebrick2")

points(USbisonrughi$SigBisonHiSPD~USbisonrughi$Date, pch="|", cex=.33, col="royalblue1")

axis(2, line=0, at=seq(0,max(USmodeldf$BisonSPD),round((max(USmodeldf$BisonSPD)/4),3)), labels=T)

mtext(2, text="Bison", line=3.5, cex=.85)

mtext(2, text="Radiocarbon Date Summed \nProbability Distributions (SPDs)", line=8, cex=.85, adj=1)

plot(1, type="n", xlab="", ylab="", axes=F, xlim=c(10000, 20000), ylim=range(USmodeldf$HumSPD)); text(10000, max(USmodeldf$HumSPD)*.95, "b."); text(19200, max(USmodeldf$HumSPD)*.95, paste("n=", datesamp$nUShum), pos=4); text(19200, (max(USmodeldf$HumSPD)*.95)-((max(USmodeldf$HumSPD)*.95)/4), paste("p<0.01"), pos=4); polygon(c(rev(USmodeldf$Date), USmodeldf$Date), c(rev(USmodeldf$HumHi), USmodeldf$HumLo), col = drkorch, border = NA); lines(USmodeldf$HumNull~USmodeldf$Date, col="darkorchid"); lines(HumSPD~Date, data=USmodeldf, type="h", col="gray50", lwd=2); points(UShumruglo$SigHumLoSPD~UShumruglo$Date, pch="|", cex=.33, col="firebrick2"); points(UShumrughi$SigHumHiSPD~UShumrughi$Date, pch="|", cex=.33, col="royalblue1"); axis(2, line=0, at=seq(0,max(USmodeldf$HumSPD),round((max(USmodeldf$HumSPD)/4),2)), labels=T); mtext(2, text="Human", line=3.5, cex=.85)

plot(1, type="n", xlab="", ylab="", axes=F, xlim=c(10000, 20000), ylim=range(GRIP2$D18O)); text(10000, max(GRIP2$D18O)*1.05, "c."); lines(GRIP2$D18O~GRIP2$BP, type="l", col="darkslateblue", lwd=2); axis(2, line=0, at=seq(min(GRIP2$D18O), max(GRIP2$D18O), 1), labels=T); mtext(2, text="Temperature", line=3.5, cex=.85)

plot(1, type="n", xlab="", ylab="", axes=F, xlim=c(10000, 20000), ylim=range(CO2.2$CO2Meanppmv)); text(10000, max(CO2.2$CO2Meanppmv)*.98, "d."); lines(CO2.2$CO2Meanppmv~CO2.2$calBP, type="l", col="darkred", lwd=2); axis(2, line=0, at=seq(min(CO2.2$CO2Meanppmv), max(CO2.2$CO2Meanppmv), 20), labels=T); mtext(2, text=expression("CO"[2]~"(ppmv)"), line=3.5, cex=.85)

plot(1, type="n", xlab="", ylab="", axes=F, xlim=c(10000, 20000), ylim=range(season2$Difference)); text(10000, max(season2$Difference)*.97, "e."); lines(season2$Difference~season2$BP, type="l", col="darkmagenta", lwd=2); axis(2, line=0, at=seq(min(season2$Difference), max(season2$Difference), 20), labels=T); mtext(2, text=expression(paste(" Seasonal \n Difference in \n Insolation (W/m"^"2"~")")), line=3.5, cex=.85)

axis(1, line=0.5, at=seq(10000,20000, by=1000), labels=c(seq(10,20,1)))

mtext(1, text="Age (ka)", line=3.75, cex=.8)

dev.off()

#########################

#Spearman's Correlations#

#########################

#Correlations between populations and time####

#Spearman's rank-order correlations between populations and time (year cal BP) express the monotonicity of populations and test for a significant rank-order relationship between populations and time

CorData <- PopData[c(20001:27101),] #Limit correlations to 20ka to the beginning of YD at 12.9ka to evaluate pre-YD population change through time

USEqDatecor <- cor.test(CorData$USEq, rev(CorData$Date), method="spearman")

USSmilDatecor <- cor.test(CorData$USSmil, rev(CorData$Date), method="spearman")

USMamDatecor <- cor.test(CorData$USMam, rev(CorData$Date), method="spearman")

USMastDatecor <- cor.test(CorData$USMast, rev(CorData$Date), method="spearman")

USNothDatecor <- cor.test(CorData$USNoth, rev(CorData$Date), method="spearman")

USHumDatecor <- cor.test(CorData$USHum, rev(CorData$Date), method="spearman")

USBisonDatecor <- cor.test(CorData$USBison, rev(CorData$Date), method="spearman")

SWMamDatecor <- cor.test(CorData$SWMam, rev(CorData$Date), method="spearman")

SWNothDatecor <- cor.test(CorData$SWNoth, rev(CorData$Date), method="spearman")

SWHumDatecor <- cor.test(CorData$SWHum, rev(CorData$Date), method="spearman")

GLMamDatecor <- cor.test(CorData$GLMam, rev(CorData$Date), method="spearman")

GLMastDatecor <- cor.test(CorData$GLMast, rev(CorData$Date), method="spearman")

GLHumDatecor <- cor.test(CorData$GLHum, rev(CorData$Date), method="spearman")

#Create data frame and export .csv file of correlation results

PopDatecor_results <- data.frame("Statistics"=c("rho","p"), "US Equus"=c(USEqDatecor$estimate, USEqDatecor$p.value), "US Smilodon"=c(USSmilDatecor$estimate, USSmilDatecor$p.value), "US Mammoths"=c(USMamDatecor$estimate, USMamDatecor$p.value), "US Mastodons"=c(USMastDatecor$estimate, USMastDatecor$p.value), "US Sloths"=c(USNothDatecor$estimate, USNothDatecor$p.value), "US Humans"=c(USHumDatecor$estimate, USHumDatecor$p.value), "US Bison"=c(USBisonDatecor$estimate, USBisonDatecor$p.value), "SW Mammoths"=c(SWMamDatecor$estimate, SWMamDatecor$p.value), "SW Sloths"=c(SWNothDatecor$estimate, SWNothDatecor$p.value), "SW Humans"=c(SWHumDatecor$estimate, SWHumDatecor$p.value), "GL Mammoths"=c(GLMamDatecor$estimate, GLMamDatecor$p.value), "GL Mastodons"=c(GLMastDatecor$estimate, GLMastDatecor$p.value), "GL Humans"=c(GLHumDatecor$estimate, GLHumDatecor$p.value))

write.csv(PopDatecor_results, "OutputTables/Population&Date_correlationresults.csv")

#Correlations between Humans and Megafauna taxa####

#Spearman's rank-order correlations between each megafauna taxon and humans test for a relationship between these two populations. If the presence or actions of humans inspired declines in megafauna populations, significant negative relationships should exist.

CorData2 <- PopData[c(25001:28300),] #limit correlations to 15ka to 11.7ka (end of YD)

#US Equus and US Humans

USeqhumcor <- cor.test(CorData2$USEq, CorData2$USHum, method="spearman")

#US Smilodon and US Humans

USsmilhumcor <- cor.test(CorData2$USSmil, CorData2$USHum, method="spearman")

#US Mammoths and US Humans

USmamhumcor <- cor.test(CorData2$USMam, CorData2$USHum, method="spearman")

#US Mastodons and US Humans

USmasthumcor <- cor.test(CorData2$USMast, CorData2$USHum, method="spearman")

#US Sloths and US Humans

USnothhumcor <- cor.test(CorData2$USNoth, CorData2$USHum, method="spearman")

#US Bison and US Humans

USbisonhumcor <- cor.test(CorData2$USBison, CorData2$USHum, method="spearman")

#SW Mammoths and SW Humans

SWmamhumcor <- cor.test(CorData2$SWMam, CorData2$SWHum, method="spearman")

#SW Sloths and SW Humans

SWnothhumcor <- cor.test(CorData2$SWNoth, CorData2$SWHum, method="spearman")

#GL Mammoths and GL Humans

GLmamhumcor <- cor.test(CorData2$GLMam, CorData2$GLHum, method="spearman")

#GL Mastodons and GL Humans

GLmasthumcor <- cor.test(CorData2$GLMast, CorData2$GLHum, method="spearman")

#Create data frame and export .csv file of correlation results

MegaHumcor_results <- data.frame("Statistics"=c("rho","p"), "US Equus/Humans"=c(USeqhumcor$estimate, USeqhumcor$p.value), "US Smilodon/Humans"=c(USsmilhumcor$estimate, USsmilhumcor$p.value), "US Mammoths/Humans"=c(USmamhumcor$estimate, USmamhumcor$p.value), "US Mastodons/Humans"=c(USmasthumcor$estimate, USmasthumcor$p.value), "US Sloths/Humans"=c(USnothhumcor$estimate, USnothhumcor$p.value), "US Bison/Humans"=c(USbisonhumcor$estimate, USbisonhumcor$p.value), "SW Mammoths/Humans"=c(SWmamhumcor$estimate, SWmamhumcor$p.value), "SW Sloths/Humans"=c(SWnothhumcor$estimate, SWnothhumcor$p.value), "GL Mammoths/Humans"=c(GLmamhumcor$estimate, GLmamhumcor$p.value), "GL Mastodons/Humans"=c(GLmasthumcor$estimate, GLmasthumcor$p.value))

write.csv(MegaHumcor_results, "OutputTables/Megafauna&Human_correlationresults_15-11.7.csv")

#####################

#Stacked Rugs Figure#

#####################

#Create vectors of values to plot the rugs stacked on top of each other----

USruglomam <- replace(USmamruglo$SigMamLoSPD, USmamruglo$SigMamLoSPD==0, 12)

USrughimam <- replace(USmamrughi$SigMamHiSPD, USmamrughi$SigMamHiSPD==0, 12)

USruglomast <- replace(USmastruglo$SigMastLoSPD, USmastruglo$SigMastLoSPD==0, 11)

USrughimast <- replace(USmastrughi$SigMastHiSPD, USmastrughi$SigMastHiSPD==0, 11)

USruglonoth <- replace(USnothruglo$SigNothLoSPD, USnothruglo$SigNothLoSPD==0, 10)

USrughinoth <- replace(USnothrughi$SigNothHiSPD, USnothrughi$SigNothHiSPD==0, 10)

USrugloeq <- replace(USeqruglo$SigEqLoSPD, USeqruglo$SigEqLoSPD==0, 9)

USrughieq <- replace(USeqrughi$SigEqHiSPD, USeqrughi$SigEqHiSPD==0, 9)

USruglosmil <- replace(USsmilruglo$SigSmilLoSPD, USsmilruglo$SigSmilLoSPD==0, 8)

USrughismil <- replace(USsmilrughi$SigSmilHiSPD, USsmilrughi$SigSmilHiSPD==0, 8)

USruglohum <- replace(UShumruglo$SigHumLoSPD, UShumruglo$SigHumLoSPD==0, 7)

USrughihum <- replace(UShumrughi$SigHumHiSPD, UShumrughi$SigHumHiSPD==0, 7)

SWruglonoth <- replace(SWnothruglo$SigNothLoSPD, SWnothruglo$SigNothLoSPD==0, 6)

SWrughinoth <- replace(SWnothrughi$SigNothHiSPD, SWnothrughi$SigNothHiSPD==0, 6)

SWruglomam <- replace(SWmamruglo$SigMamLoSPD, SWmamruglo$SigMamLoSPD==0, 5)

SWrughimam <- replace(SWmamrughi$SigMamHiSPD, SWmamrughi$SigMamHiSPD==0, 5)

SWruglohum <- replace(SWhumruglo$SigHumLoSPD, SWhumruglo$SigHumLoSPD==0, 4)

SWrughihum <- replace(SWhumrughi$SigHumHiSPD, SWhumrughi$SigHumHiSPD==0, 4)

GLruglomast <- replace(GLmastruglo$SigMastLoSPD, GLmastruglo$SigMastLoSPD==0, 3)

GLrughimast <- replace(GLmastrughi$SigMastHiSPD, GLmastrughi$SigMastHiSPD==0, 3)

GLruglomam <- replace(GLmamruglo$SigMamLoSPD, GLmamruglo$SigMamLoSPD==0, 2)

GLrughimam <- replace(GLmamrughi$SigMamHiSPD, GLmamrughi$SigMamHiSPD==0, 2)

GLruglohum <- replace(GLhumruglo$SigHumLoSPD, GLhumruglo$SigHumLoSPD==0, 1)

GLrughihum <- replace(GLhumrughi$SigHumHiSPD, GLhumrughi$SigHumHiSPD==0, 1)

#plot stacked rugs----

jpeg("Figures/StackedRugs.jpeg", width = 7, height = 6, units = 'in', res = 300)

par(mar=c(5,9,3,2) + 0.1, xpd=F, mfrow=c(1,1))

plot(1, type="n", xlim=c(11000,14000), ylim=c(0.75,12.25), xlab="Cal BP", ylab="", yaxt="n")

rect(11650, -1, 12900, 13, border=F, col="cadetblue1")

rect(12900, -1, 13200, 13, border=F, col="orange")

par(xpd=NA)

text(12300, 12.75, srt=0, pos=3, cex=.75, "Younger Dryas")

text(13050, 12.75, srt=0, pos=3, cex=.75, "Clovis")

par(xpd=F)

points(USruglomam~USmamruglo$Date, pch="|", cex=.33, col="firebrick2")

points(USrughimam~USmamrughi$Date, pch="|", cex=.33, col="royalblue1")

points(USruglomast~USmastruglo$Date, pch="|", cex=.33, col="firebrick2")

points(USrughimast~USmastrughi$Date, pch="|", cex=.33, col="royalblue1")

points(USruglonoth~USnothruglo$Date, pch="|", cex=.33, col="firebrick2")

points(USrughinoth~USnothrughi$Date, pch="|", cex=.33, col="royalblue1")

points(USrugloeq~USeqruglo$Date, pch="|", cex=.33, col="firebrick2")

points(USrughieq~USeqrughi$Date, pch="|", cex=.33, col="royalblue1")

points(USruglosmil~USsmilruglo$Date, pch="|", cex=.33, col="firebrick2")

points(USrughismil~USsmilrughi$Date, pch="|", cex=.33, col="royalblue1")

points(USruglohum~UShumruglo$Date, pch="|", cex=.33, col="firebrick2")

points(USrughihum~UShumrughi$Date, pch="|", cex=.33, col="royalblue1")

points(SWruglonoth~SWnothruglo$Date, pch="|", cex=.33, col="firebrick2")

points(SWrughinoth~SWnothrughi$Date, pch="|", cex=.33, col="royalblue1")

points(SWruglomam~SWmamruglo$Date, pch="|", cex=.33, col="firebrick2")

points(SWrughimam~SWmamrughi$Date, pch="|", cex=.33, col="royalblue1")

points(SWruglohum~SWhumruglo$Date, pch="|", cex=.33, col="firebrick2")

points(GLruglomast~GLmastruglo$Date, pch="|", cex=.33, col="firebrick2")

points(GLrughimast~GLmastrughi$Date, pch="|", cex=.33, col="royalblue1")

points(GLruglomam~GLmamruglo$Date, pch="|", cex=.33, col="firebrick2")

points(GLrughimam~GLmamrughi$Date, pch="|", cex=.33, col="royalblue1")

points(GLruglohum~GLhumruglo$Date, pch="|", cex=.33, col="firebrick2")

points(GLrughihum~GLhumrughi$Date, pch="|", cex=.33, col="royalblue1")

axislab <- c("US Mammoth", "US Mastodon", "US Sloth", "US Equus", "US Smilodon", "US Human", "SW Sloth", "SW Mammoth", "SW Human", "GL Mastodon", "GL Mammoth", "GL Human")

axis(side=2, at=seq(1,12), labels=rev(axislab), las=2)

box()

dev.off()

**R Code for Taphonomic Correction Supplement**

#Run the same code above but swap the original spd() and modelTest() functions for the revised functions below:

#Create a version of the spd() function from the rcarbon package which taphonomically corrects the resulting SPD

taphspd <- function(x, timeRange, bins=NA, datenormalised=FALSE, spdnormalised=FALSE, runm=NA, verbose=TRUE){

defcall <- as.list(args(spd))

defcall <- defcall[-length(defcall)]

speccall <- as.list(match.call())

speccall <- speccall[-1]

i <- match(names(defcall), names(speccall))

i <- is.na(i)

if (any(i)){

speccall[names(defcall)[which(i)]] <- defcall[which(i)]

}

speccall <- as.data.frame(lapply(speccall,deparse), stringsAsFactors=FALSE)

speccall <- speccall[,names(defcall)]

speccall$ndates <- nrow(x$metadata)

speccall$nbins <- nrow(x$metadata)

if (!"CalDates" %in% class(x)){

stop("x must be an object of class 'CalDates'.")

}

if (length(bins)>1){

speccall$nbins <- length(unique(bins))

if (any(is.na(bins))){

stop("Cannot have NA values in bins.")

}

if (length(bins)!=nrow(x$metadata)){

stop("bins (if provided) must be the same length as x.")

}

} else {

bins <- rep("0_0",nrow(x$metadata))

}

binNames <- unique(bins)

calyears <- data.frame(calBP=seq(timeRange[1], timeRange[2],-1))

binnedMatrix <- matrix(NA, nrow=nrow(calyears), ncol=length(binNames))

if (verbose){

if (length(x$calmatrix)>1){

print("Aggregating...")

} else {

print("Extracting and aggregating...")

}

}

if (verbose & length(binNames)>1){

flush.console()

pb <- txtProgressBar(min=1, max=length(binNames), style=3)

}

caldateTR <- as.numeric(x$metadata[1,c("StartBP","EndBP")])

caldateyears <- seq(caldateTR[1],caldateTR[2],-1)

check <- caldateTR[1] >= timeRange[1] & caldateTR[2] <= timeRange[2]

for (b in 1:length(binNames)){

if (verbose & length(binNames)>1){ setTxtProgressBar(pb, b) }

index <- which(bins==binNames[b])

if (length(x$calmatrix)>1){

if (!check){

stop("The time range of the calibrated dataset must be at least as large as the spd time range.")

} else {

tmp <- x$calmatrix[,index, drop=FALSE]

if (datenormalised){

tmp <- apply(tmp,2,FUN=function(x) x/sum(x))

}

spdtmp <- rowSums(tmp)

if (length(binNames)>1){

spdtmp <- spdtmp / length(index)

}

binnedMatrix[,b] <- spdtmp[caldateyears<=timeRange[1] & caldateyears>=timeRange[2]]

}

} else {

slist <- x$grids[index]

slist <- lapply(slist,FUN=function(x) merge(calyears,x, all.x=TRUE))

slist <- rapply(slist, f=function(x) ifelse(is.na(x),0,x), how="replace")

slist <- lapply(slist, FUN=function(x) x[with(x, order(-calBP)), ])

tmp <- lapply(slist,`[`,2)

if (datenormalised){

outofTR <- lapply(tmp,sum)==0 # date out of range

tmpc <- tmp[!outofTR]

if (length(tmpc)>0){

tmp <- lapply(tmpc,FUN=function(x) x/sum(x))

}

}

if (length(binNames)>1){

spdtmp <- Reduce("+", tmp) / length(index)

} else {

spdtmp <- Reduce("+", tmp)

}

binnedMatrix[,b] <- spdtmp[,1]

}

}

if (verbose & length(binNames)>1){ close(pb) }

finalSPD <- apply(binnedMatrix,1,sum)

if (!is.na(runm)){

finalSPD <- runMean(finalSPD, runm, edge="fill")

}

#Add taphonomic correction according to Equation 1 in Surovell et al. (2009)

lostpop<-(5.726442*(10^6*(calyears$calBP+2176.4)^-1.3925309))

res <- data.frame(calBP=calyears$calBP, PrDens=((finalSPD/lostpop)*max(finalSPD)))

if (spdnormalised){

res$PrDens <- res$PrDens/sum(res$PrDens, na.rm=TRUE)

}

res <- res[res$calBP <= timeRange[1] & res$calBP >= timeRange[2],]

class(res) <- c("CalGrid", class(res))

reslist <- vector("list",length=2)

names(reslist) <- c("metadata","grid")

reslist[["metadata"]] <- speccall

reslist[["grid"]] <- res

class(reslist) <- c("CalSPD",class(reslist))

if (verbose){ print("Done.") }

return(reslist)

}

#Create a version of the modelTest() function from the rcarbon package which taphonomically corrects the SPD used

modelTest.taph <- function(x, errors, nsim, bins=NA, runm=NA, timeRange=NA, raw=FALSE, model=c("exponential","explog","custom"), predgrid=NA, calCurves='intcal13', datenormalised=FALSE, spdnormalised=FALSE, ncores=1, fitonly=FALSE, a=0, b=0, verbose=TRUE){

if (ncores>1&!requireNamespace("doParallel", quietly=TRUE)){

warning("the doParallel package is required for multi-core processing; ncores has been set to 1")

ncores=1

} else {

cl <- parallel::makeCluster(ncores)

doParallel::registerDoParallel(cl)

on.exit(stopCluster(cl))

}

if (verbose){ print("Aggregating observed dates...") }

if (is.na(bins[1])){

samplesize <- nrow(x$metadata)

} else {

samplesize <- length(unique(bins))

}

observed <- spd(x=x, bins=bins, timeRange=timeRange, datenormalised=datenormalised, runm=runm, spdnormalised=spdnormalised, verbose=FALSE)

#Add taphonomic correction according to Equation 1 in Surovell et al (2009)

lostpop<-(5.726442*(10^6*(observed$grid$calBP+2176.4)^-1.3925309))

finalSPD <- ((observed$grid$PrDens/lostpop)*max(observed$grid$PrDens))

if (fitonly == TRUE) {nsim <- 1}

## Simulation

sim <- matrix(NA,nrow=length(finalSPD),ncol=nsim)

if (verbose & !fitonly){

print("Monte-Carlo test...")

flush.console()

pb <- txtProgressBar(min=1, max=nsim, style=3)

}

time <- seq(timeRange[1],timeRange[2],-1)

fit <- NA

if (model=="exponential"){

fit <- nls(y ~ exp(a + b * x), data=data.frame(x=time, y=finalSPD), start=list(a=a, b=b))

est <- predict(fit, list(x=time))

predgrid <- data.frame(calBP=time, PrDens=est)

} else if (model=="uniform"){

predgrid <- data.frame(calBP=time, PrDens=mean(finalSPD))

} else if (model=="linear"){

fit <- lm(y ~ x, data=data.frame(x=time, y=finalSPD))

est <- predict(fit, list(x=time))

predgrid <- data.frame(calBP=time, PrDens=est)

} else if (model=="custom"){

if (length(predgrid)!=2){

stop("If you choose a custom model, you must provide a proper predgrid argument (two-column data.frame of calBP and predicted densities).")

}

} else {

stop("Specified model not one of current choices.")

}

if (fitonly){

print("Done (SPD and fitted model only).")

res <- list(result=NA, sim=NA, pval=NA, osbSPD=observed, fit=predgrid, fitobject=fit)

return(res)

}

cragrid <- uncalibrate(as.CalGrid(predgrid), calCurves=calCurves, compact=FALSE, verbose=FALSE)

cragrid <- cragrid[cragrid$CRA <= max(x$metadata$CRA) & cragrid$CRA >= min(x$metadata$CRA),]

if (ncores==1)

{

for (s in 1:nsim){

if (verbose){ setTxtProgressBar(pb, s) }

randomDates <- sample(cragrid$CRA, replace=TRUE, size=samplesize, prob=cragrid$PrDens)

randomSDs <- sample(size=length(randomDates), errors, replace=TRUE)

tmp <- calibrate(x=randomDates,errors=randomSDs, timeRange=timeRange, calCurves=calCurves, normalised=datenormalised, ncores=1, verbose=FALSE, calMatrix=TRUE)

simDateMatrix <- tmp$calmatrix

sim[,s] <- apply(simDateMatrix,1,sum)

sim[,s] <- (sim[,s]/sum(sim[,s])) * sum(predgrid$PrDens[predgrid$calBP <= timeRange[1] & predgrid$calBP >= timeRange[2]])

if (spdnormalised){ sim[,s] <- (sim[,s]/sum(sim[,s])) }

if (!is.na(runm)){ sim[,s] <- runMean(sim[,s], runm, edge="fill") }

}

}

if (ncores>1)

{

print("Progress bar disabled for multi-core processing")

sim <- foreach (s = 1:nsim, .combine='cbind', .packages='rcarbon') %dopar% {

# if (verbose){ setTxtProgressBar(pb, s) }

randomDates <- sample(cragrid$CRA, replace=TRUE, size=samplesize, prob=cragrid$PrDens)

randomSDs <- sample(size=length(randomDates), errors, replace=TRUE)

tmp <- calibrate(x=randomDates,errors=randomSDs, timeRange=timeRange, calCurves=calCurves, normalised=datenormalised, ncores=1, verbose=FALSE, calMatrix=TRUE)

simDateMatrix <- tmp$calmatrix

aux <- apply(simDateMatrix,1,sum)

aux <- (aux/sum(aux)) * sum(predgrid$PrDens[predgrid$calBP <= timeRange[1] & predgrid$calBP >= timeRange[2]])

if (spdnormalised){ aux <- (aux/sum(aux)) }

if (!is.na(runm)){

aux <- runMean(aux, runm, edge="fill")

}

aux

}

#stopCluster(cl)

}

if (verbose){ close(pb) }

## Envelope, z-scores, global p-value

lo <- apply(sim,1,quantile,prob=0.025)

hi <- apply(sim,1,quantile,prob=0.975)

Zsim <- t(apply(sim,1,scale))

zLo <- apply(Zsim,1,quantile,prob=0.025,na.rm=TRUE)

zHi <- apply(Zsim,1,quantile,prob=0.975,na.rm=TRUE)

Zscore_empirical <- (finalSPD - apply(sim, 1, mean))/apply(sim, 1, sd)

busts <- which(Zscore_empirical< zLo)

booms <- which(Zscore_empirical> zHi)

busts2 <- which(finalSPD< lo)

booms2 <- which(finalSPD> hi)

observedStatistic <- sum(c(zLo[busts] - Zscore_empirical[busts]),c(Zscore_empirical[booms]-zHi[booms]))

expectedstatistic <- abs(apply(Zsim,2,function(x,y){a=x-y;i=which(a<0);return(sum(a[i]))},y=zLo)) + apply(Zsim,2,function(x,y){a=x-y;i=which(a>0);return(sum(a[i]))},y=zHi)

pvalue <- 1 - c(length(expectedstatistic[expectedstatistic <= observedStatistic]))/c(length(expectedstatistic)+1)

# Results

result <- data.frame(calBP=observed$grid$calBP,PrDens=finalSPD,lo=lo,hi=hi)

if(raw==FALSE){ sim <- NA }

res <- list(result=result, sim=sim, pval=pvalue, fit=predgrid, fitobject=fit)

class(res) <- "SpdModelTest"

if (verbose){ print("Done.") }

return(res)

}
