# Supplementary material for: Population reconstructions for humans and megafauna suggest mixed causes for North American Pleistocene extinctions
Source: Nat Commun. 2018 Dec 21;9:5441. doi: 10.1038/s41467-018-07897-1 (PMC6303330; doi:10.1038/s41467-018-07897-1)
Supplement: Supplementary file 6 — Reporting Summary [file 41467_2018_7897_MOESM6_ESM.pdf]

nature research

Corresponding author(s): *Double-blind peer review submissions: write DBPR and your manuscript number here instead of author names.*

Reporting Summary

Nature Research wishes to improve the reproducibility of the work that we publish. This form provides structure for consistency and transparency in reporting. For further information on Nature Research policies, see Authors & Referees and the Editorial Policy Checklist.

Statistical parameters

When statistical analyses are reported, confirm that the following items are present in the relevant location (e.g. figure legend, table legend, main text, or Methods section).

n/a	Confirmed	
☐	☒	The exact sample size (n) for each experimental group/condition, given as a discrete number and unit of measurement
☐	☒	An indication of whether measurements were taken from distinct samples or whether the same sample was measured repeatedly
☐	☒	The statistical test(s) used AND whether they are one- or two-sided<br>*Only common tests should be described solely by name; describe more complex techniques in the Methods section.*
☒	☐	A description of all covariates tested
☒	☐	A description of any assumptions or corrections, such as tests of normality and adjustment for multiple comparisons
☒	☐	A full description of the statistics including central tendency (e.g. means) or other basic estimates (e.g. regression coefficient) AND variation (e.g. standard deviation) or associated estimates of uncertainty (e.g. confidence intervals)
☐	☒	For null hypothesis testing, the test statistic (e.g. F, t, r) with confidence intervals, effect sizes, degrees of freedom and P value noted<br>*Give P values as exact values whenever suitable.*
☒	☐	For Bayesian analysis, information on the choice of priors and Markov chain Monte Carlo settings
☒	☐	For hierarchical and complex designs, identification of the appropriate level for tests and full reporting of outcomes
☒	☐	Estimates of effect sizes (e.g. Cohen's d, Pearson's r), indicating how they were calculated
☐	☒	Clearly defined error bars<br>*State explicitly what error bars represent (e.g. SD, SE, CI)*

Our web collection on statistics for biologists may be useful.

Software and code

Policy information about availability of computer code

Data collection	No commercial open source or custom code was used to collect the data in this study
Data analysis	R version 3.5.0 was used in the analysis of the data. All code was written by the authors and is provided as Supplementary Data 2. The background map of the states and provinces in Figure 1 was downloaded using Environmental Systems Research Institute (ESRI). ArcGIS Desktop Release 10.5.1. Redlands, CA (2017)— that reference is cited in the Figure 1 caption in the text and included in the references cited (reference 59).

For manuscripts utilizing custom algorithms or software that are central to the research but not yet described in published literature, software must be made available to editors/reviewers upon request. We strongly encourage code deposition in a community repository (e.g. GitHub). See the Nature Research guidelines for submitting code & software for further information.

Data

Policy information about availability of data

All manuscripts must include a data availability statement. This statement should provide the following information, where applicable:

- Accession codes, unique identifiers, or web links for publicly available datasets
- A list of figures that have associated raw data
- A description of any restrictions on data availability

All megafauna radiocarbon data used are from previously published sources and are listed in Supplementary Data 1. All human radiocarbon data are provided from the CARD: Canadian Archaeological Radiocarbon Database (CARD 2.1) Geospatial Radiocarbon Data (Accessed 15 May 2017). See http://www.canadianarchaeology.ca/

Field-specific reporting

Please select the best fit for your research. If you are not sure, read the appropriate sections before making your selection.

☐ Life sciences ☐ Behavioural & social sciences ☒ Ecological, evolutionary & environmental sciences

For a reference copy of the document with all sections, see nature.com/authors/policies/ReportingSummary-flat.pdf

Ecological, evolutionary & environmental sciences study design

All studies must disclose on these points even when the disclosure is negative.

Study description	Human and megafauna populations were reconstructed based on radiocarbon date frequencies (Summed Probability Distributions)—significant negative correlations would suggest human hunting caused population declines. Spearman's rank order correlation was used to evaluate such correlations. We compared each SPD to a null model to identify periods of time where the SPD deviates significantly from expected values. The null model to which we compared each SPD represents the trajectory each megafaunal taxon or human SPD followed prior to its decline. To create such a null model, we first used segmented regression analysis to fit paired quasi-Poisson family generalized linear models (GLMs) with a log link function to the SPD using the segmented package70. This analysis identified “breakpoints” at which the two GLMs were joined. These breakpoints represent the date at which the SPD shifts from an overall pattern of increasing to decreasing values. We next created a single quasi-Poisson family GLM with a log link that was based on the SPD values from 20.0 ka to the taxon’s specific breakpoint date, thereby representing only the increasing portion of the SPD. This analysis allowed us to determine the timing of population declines leading to extinction.
Research sample	All available previously published direct radiocarbon dates on megafauna from the contiguous United States were used in the study. All human radiocarbon dates from the CARD (described above) from the contiguous United States were used in the study
Sampling strategy	All available samples from the contiguous United States were used in the study; no sampling of available data was conducted.
Data collection	Broughton reviewed the literature to retrieve the direct radiocarbon dates on megafauna. Weitzel queried the CARD to access all available human radiocrabon dates.
Timing and spatial scale	Data collected were from previously published sources; no field or experimental analyses were conducted to obtain data.
Data exclusions	No data were excluded from the analysis.
Reproducibility	No experiments were conducted.
Randomization	All available dates from megafauna and humans were utilized: no sampling was conducted as part of the study.
Blinding	No experiments were conducted in the study

Did the study involve field work? ☐ Yes ☒ No

Reporting for specific materials, systems and methods

Materials & experimental systems

n/a	Involved in the study
☒	☐ Unique biological materials
☒	☐ Antibodies
☒	☐ Eukaryotic cell lines
☐	☒ Palaeontology
☒	☐ Animals and other organisms
☒	☐ Human research participants

Methods

n/a	Involved in the study
☒	☐ ChIP-seq
☒	☐ Flow cytometry
☒	☐ MRI-based neuroimaging

Palaeontology

Specimen provenance	No specimens were collected as part of this study
Specimen deposition	No specimens were collected as part of this study
Dating methods	No specimens were collected as part of this study

☒ Tick this box to confirm that the raw and calibrated dates are available in the paper or in Supplementary Information.
